# Inferring disease course from differential exon usage in the wide titinopathy spectrum

**DOI:** 10.1002/acn3.52189

**Published:** 2024-08-28

**Authors:** Maria Francesca Di Feo, Ali Oghabian, Ella Nippala, Mathias Gautel, Heinz Jungbluth, Francesca Forzano, Edoardo Malfatti, Claudia Castiglioni, Ilona Krey, David Gomez Andres, Angela F. Brady, Maria Iascone, Anna Cereda, Lidia Pezzani, Daniel Natera De Benito, Andres Nascimiento Osorio, Berta Estévez Arias, Sergei A. Kurbatov, Tania Attie‐Bitach, Sheela Nampoothiri, Erin Ryan, Michelle Morrow, Svetlana Gorokhova, Brigitte Chabrol, Juha Sinisalo, Heli Tolppanen, Johanna Tolva, Francina Munell, Jessica Camacho Soriano, Maria Angeles Sanchez Duran, Mridul Johari, Homa Tajsharghi, Peter Hackman, Bjarne Udd, Marco Savarese

**Affiliations:** ^1^ Department of Neuroscience, Rehabilitation, Ophthalmology, Genetics, and Maternal and Child Health (DINOGMI) University of Genoa Genoa Italy; ^2^ Folkhälsan Research Center Helsinki Uusimaa Finland; ^3^ Randall Division of Cell and Molecular Biophysics and Cardiovascular Division King's College London BHF Centre of Research Excellence London UK; ^4^ Paediatric Neurology Neuromuscular Service, Evelina's Children Hospital, Guy's and St Thomas' Hospitals NHS Trust London UK; ^5^ Clinical Genetics Department Guy's and St Thomas NHS Foundation Trust London SE1 9RT UK; ^6^ Université Paris Est Créteil, INSERM, U955, IMRB, and Reference Center for Neuromuscular Disorders, APHP Henri Mondor University Hospital Créteil France; ^7^ Clinica MEDS Santiago de Chile Chile; ^8^ Institute of Human Genetics, University of Leipzig Hospitals and Clinics Leipzig 4275 Germany; ^9^ Child Neurology Unit. Hospital Universitari Vall d'Hebron, Vall d'Hebron Research Institute (VHIR) Barcelona Spain; ^10^ North West Thames Regional Service, Northwick Park and St. Mark's Hospitals Harrow London UK; ^11^ Medical Genetics Laboratory ASST Papa Giovanni XXIII Bergamo Italy; ^12^ Clinical Genetics Service Pediatria 1—ASST Papa Giovanni XXIII Bergamo Italy; ^13^ Neuropaediatrics Department Hospital Sant Joan De Déu, Institut De Recerca Sant Joan De Déu Barcelona 08950 Spain; ^14^ Neuromuscular Unit Department of Neurology, Hospital Sant Joan De Déu Barcelona Spain; ^15^ Voronezh NN Burdenko State Medical University Voronezh 394036 Russia; ^16^ Saratov State Medical University Saratov 410012 Russia; ^17^ Unité D'embryofoetopathologie Service D'histologie‐Embryologie‐Cytogénétique, Hôpital Necker‐Enfants Malades Paris France; ^18^ Department of Pediatric Genetics Amrita Institute of Medical Sciences & Research Centre Kochi Kerala India; ^19^ GeneDx Gaithersburg Maryland USA; ^20^ Marseille Medical Genetics Aix Marseille Université, Faculté Des Sciences Médicales Et Paramédicales Marseille France; ^21^ Reference Center for Inherited Metabolic Diseases Marseille University Hospital Marseille France; ^22^ Helsinki University Central Hospital Helsinki Finland; ^23^ Transplantation Laboratory, Department of Pathology University of Helsinki Helsinki Finland; ^24^ Unitat De Malalties Neuromusculars Pediàtriques Hospital Universitari Vall D'Hebron Barcelona Spain; ^25^ Histology Department Vall D'Hebron University Hospital Barcelona Spain; ^26^ Maternal Fetal Medicine Unit, Department of Obstetrics Universitat Autònoma de Barcelona, Hospital Vall D'Hebron Barcelona Spain; ^27^ Harry Perkins Institute of Medical Research, Centre for Medical Research University of Western Australia Nedlands Western Australia Australia; ^28^ Division of Biomedicine, School of Health Sciences University of Skovde Skovde Sweden; ^29^ Department of Musculoskeletal Diseases Tampere University Hospital Tampere Pirkanmaa Finland

## Abstract

**Objective:**

Biallelic titin truncating variants (TTNtv) have been associated with a wide phenotypic spectrum, ranging from complex prenatal muscle diseases with dysmorphic features to adult‐onset limb‐girdle muscular dystrophy, with or without cardiac involvement. Given the size and complexity of TTN, reaching an unequivocal molecular diagnosis and precise disease prognosis remains challenging.

**Methods:**

In this case series, 12 unpublished cases and one already published case with biallelic TTNtv were collected from multiple international medical centers between November 2022 and September 2023. TTN mutations were detected through exome or genome sequencing. Information about familial and personal clinical history was collected in a standardized form. RNA‐sequencing and analysis of TTN exon usage were performed on an internal sample cohort including postnatal skeletal muscles, fetal skeletal muscles, postnatal heart muscles, and fetal heart muscles. In addition, publicly available RNA‐sequencing data was retrieved from ENCODE.

**Results:**

We generated new RNA‐seq data on TTN exons and identified genotype–phenotype correlations with prognostic implications for each titinopathy patient (whether worsening or improving in prenatal and postnatal life) using percentage spliced in (PSI) data for the involved exons. Interestingly, thanks to exon usage, we were also able to rule out a titinopathy diagnosis in one prenatal case.

**Interpretation:**

This study demonstrates that exon usage provides valuable insights for a more exhaustive clinical interpretation of TTNtv; additionally, it may serve as a model for implementing personalized medicine in many other genetic diseases, since most genes undergo alternative splicing.

## Introduction

*TTN* gene encodes for the giant protein titin, which has a crucial role in sarcomere development, structure, signaling, and myofibrillar stability during muscle contraction‐relaxation.^1^, ^2^ It contains 363 coding exons plus the first noncoding exon, which are all contained in the inferred theoretical complete “metatranscript” isoform, identified by the conventional *TTN* MANE Select reference transcript (NM_001267550.2).^3^

As expected, titin has a very complex splicing pattern, with more than 1 million splice variants potentially generated.^4^, ^5^ Overall, the diversity of titin isoforms produced by alternative splicing is thought to contribute to the complexity and adaptability of muscle function, and to the variability of disease involvement of anatomically distinct muscles.^6^, ^7^

Considering this variability, the position of *TTN* variants is crucial to correlate the molecular findings with the clinical phenotypes. For example, heterozygous truncating variants in cardiac isoforms (with the principle long cardiac isoform being N2BA) have been associated with dilated cardiomyopathy (DCM), while truncating variants in other regions do not necessarily imply cardiac risk.^8^, ^9^, ^10^ Similarly, variants in the last canonical exon (364) are associated with dominant tibial muscular dystrophy, whereas biallelic TTNtv in the two last exons with juvenile‐early adult‐onset recessive distal titinopathy.^11^, ^12^, ^13^ Variants in exon 344 cause usually dominant hereditary myopathy with early respiratory failure (HMERF).^14^ However, to date, our understanding of the role of *TTN* exons and their differential expression throughout developmental stages and tissues has been partial, so much as it appears we are barely scratching the surface.

Biallelic truncating variants in metatranscript‐only exons have been associated with prenatal and congenital severe myopathies.^15^, ^16^, ^17^ Remarkably, for some patients with severe respiratory and feeding issues at birth, the differential diagnosis includes a wide range of congenital disorders.^18^ Also, the most severe congenital titinopathies resemble syndromic phenotypes, affecting not only muscles but also bone, heart, and other organs, with a quite high rate of dysmorphisms (up to 36%).^19^ Notably, brain abnormalities presenting both in prenatal and postnatal life have been described in a few published cases, although the presence of pathogenic variants in other genes cannot be excluded.^18^, ^20^, ^21^

Traditionally, metatranscript‐only exons are not supposed to be expressed in postnatal muscles, while, on the other side, canonical exons are supposed to have a high expression both in fetal and postnatal skeletal muscles. However, clinical reports published in recent years suggest that this may not always be the case.^17^, ^22^, ^23^

In our previous significant effort, we developed specific recommendations for *TTN* variants interpretation.^24^ Considering our increased knowledge of the phenotypic spectrum and the crucial insights provided using RNA‐sequencing techniques, we propose a new comprehensive workflow for the clinical interpretation of genetic findings in *TTN*, which relies on the current evidence on exon usage.

## Material and Methods

### Clinical features analysis

We collected biallelic titinopathy cases that have been brought to our attention by direct request for counseling and one previously published case. In particular, the unpublished cases have been selected by geneticists from different international centers for carrying biallelic TTNtv. In order to avoid possible biases, we limited the study to cases with biallelic variants causing a premature stop codon (nonsense and indels causing a frameshift), excluding patients with variants predicted to alter the splicing.

All the cases have been clinically assessed either by gynecologists experienced in prenatal diagnostics for prenatal cases, by child neurologists for children, and by neuromuscular‐expert neurologists for adults. We collected all the clinical data, including prenatal and family history information, in a standardized form (Table S1). Regarding the already published case, we collected extra details from the manuscript's authors.

### Molecular genetic analysis

Proband DNA was analyzed using short‐read exome (ES) or genome (GS) sequencing in each referring institution. Sequencing data were analyzed using standard bioinformatic pipelines aiming at the identification of single nucleotide variants, small insertions or deletion (indels). All the recruited patients carried biallelic TTNtv. Segregation analysis was performed to confirm the phase of the variants.

### RNA‐sequencing on samples cohort

We performed RNA‐sequencing on a large samples cohort to determine the ratio at which each titin exon is included (i.e., not spliced out) among transcripts from four different tissue types (postnatal skeletal, postnatal cardiac, fetal skeletal, and fetal cardiac). For postnatal skeletal muscle analysis, we collected muscle samples dissected from both myopathic and non‐myopathic individuals (41 individuals aging 0–89 years from different international centers) (Supplemental Material and Methods, Figure S1, Table S2).

Also, seven heart muscles samples (left ventricle) dissected from adult individuals that have undergone transplantation for ischemic heart disease were included (collected at the Department of Pathology, University of Helsinki, Finland). Fetal expression analysis was performed using publicly available database (ENCODE). Fetal skeletal muscles (*n* = 20) and fetal heart muscles (*n* = 2) from two different fetuses, without muscle pathology, were obtained from voluntary TOPs (collected at the Hospital Universitari Vall D'Hebron, Barcelona, Spain).

For long‐read sequencing, data were generated from five different skeletal muscles and one fetal heart muscle, belonging to the same fetus.

The paired‐end RNAseq reads were aligned using the splice‐aware STAR alignment software (version 2.7.7a).^25^ The Percentage Spliced In (PSI) values of *TTN* exons were measured using the Intron Exon Retention Estimator (IntEREst) R/Bioconductor package (V1.24.0).^26^ For long reads, transcriptome analysis was performed with the SQANTI software with default parameters.^27^ Only *TTN* transcripts (Ensembl ID ENSG00000155657) were included in the analysis and duplicates were filtered out. The extracted sequences were aligned and identified using the BLAST‐like Alignment tool (BLAT).

## Results

We report 13 patients with biallelic truncating variants in the *TTN* gene; five out of 13 carry biallelic TTNtv in exons considered as “canonical” by the current classifications. To explain the clinical phenotype, we attempted to correlate signs and symptoms with exon usage data obtained by RNA‐seq analysis.

The clinical details of each titinopathy patient are summarized in Tables 1 and 2 and described in greater detail in Table S1.

**Table 1:** Clinical summary of the patients with biallelic TTNtv in compound heterozygosity and exon usage associated to each variant.

ID	Clinical category	Prenatal signs and symptoms	Postnatal signs and symptoms	Improved after birth	Variant 1			Variant 2		
					Annotation (exon)	Exon usage in fetal SM (%)	Exon usage in adult SM (%)	Annotation (exon)	Exon usage in fetal SM (%)	Exon usage in adult SM (%)
P1	Deceased before birth (TOP)	IUGR, amyoplasia, hydrops, arthrogryposis	NA	NA	c.14183dup (50)	99	97	c.35182_35188del (156)	70	26
P2	Deceased before birth (fetal death)	Amyoplasia, arthrogryposis	NA	NA	c.23386C>T (82)	95	86	c.34408del (149)	55	25
P3	Deceased after birth	Arthrogryposis, amyoplasia, dysmorphisms	Respiratory insufficiency	NA	c.85348A>T (327)	97	93	c.36100_36101del (167)	63	4
P5	Improving with age (infant)	Fetal akinesia, arthrogryposis	Hypotonia, respiratory insufficiency, dysphagia, congenital fractures	Yes	c.96464del (348)	94	90	c.36040A>T (166)	39	1
P6	Improving with age (child)	Arthrogryposis	Hypotonia, respiratory insufficiency, dysphagia, congenital fractures	Yes	c.33055del (136)	77	51	c.38737G>T (199)	15	2
P7	Improving with age (adult)	Arthrogryposis	Hypotonia, proximal and distal weakness, reduced vital capacity	Yes, independent walking acquired	c.67495C>T (320)	92	84	c.38737G>T (199)	15	2
P8	Improving with age (adult)	Arthrogryposis	Hypotonia, proximal and distal weakness	Yes, walking with aids	c.103531A>T (359)	94	94	c.38661_38665del (198)	11	1
P10	Worsening with age	Not reported	Hypotonia, sluggish reflex	No, worsened, respiratory insufficiency at 18 months, motor delay, concentric hypertrophic cardiomyopathy	c.70978C>T (327)	97	93	c.2047C>T (13)	31	68
P11	Worsening with age (death at 6 months)	Arthrogryposis, threatened miscarriage	Hypotonia, respiratory insufficiency, dysphagia	No, worsened, death at 6 months of age	c.65163T>A (312)	92	87	c.32680del (133)	21	30
P13	Unlikely case of titinopathy, TOP	Macrocephaly >90 + p, vertebral fusion D7‐D8‐D9, multicystic lymphangioma of the mesentery	NA	NA	c.55939G>T (289)	96	93	c.10439dupA (45)	4	0

IUGR, intrauterine growth restriction; SM, skeletal muscles; TOP, termination of pregnancy.

**Table 2:** Clinical summary of the patients with biallelic TTNtv in homozygosity and exon usage associated to each variant.

ID	Clinical category	Prenatal signs and symptoms	Postnatal signs and symptoms	Improved after birth	Variant 1			Variant 2		
					Annotation (exon)	Exon usage in fetal SM (%)	Exon usage in adult SM (%)	Annotation (exon)	Exon usage in fetal SM (%)	Exon usage in adult SM (%)
P4	Improving with age (infant)	IUGR, amyoplasia, arthrogryposis	Hypotonia, respiratory insufficiency, dysphagia, congenital fractures	Yes	c.40267G>T (217)	68	27	c.40267G>T (217)	68	27
P9	Improving with age (adult)	No	Hypotonia, distal arthrogryposis	Yes	c.38661_38665del (198)	11	1	c.38661_38665del (198)	11	1
P12	Worsening with age (adolescent)	No	HyperCKemia at 14 years of age	No	c.32656C>T (133)	21	30	c.32656C>T (133)	21	30

SM, skeletal muscles.

### Patients deceased before birth or in the perinatal period

Three cases (P1–P3) were unborn fetuses: P1 and P2 underwent termination of pregnancy because of severe prenatal findings such as intrauterine growth restriction (IUGR), fetal akinesia with hypo/amyoplasia and hydrops. P3 died in the first hour after birth. They all presented with arthrogryposis, fetal akinesia, and hypotonia and P3 also had unspecific facial dysmorphisms.

P1–P3 are compound heterozygous for TTNtv. They all carry one TTNtv located in an A‐band or I‐band exon highly expressed in skeletal muscles in all developmental stages (PSI 85%–95%), and another TTNtv on the other allele, located in a more variably expressed exon.

Interestingly, two of them P1 and P2 carry the second variant in canonical exons 156 and 149, which have a PSI of 26% and 25% in postnatal skeletal muscle, respectively, but 70% and 55%, respectively, in fetal muscles. Patient 3 carries a second TTNtv in exon 167, a metatranscript only exon with a high PSI in fetal muscles (63%) and a barely detectable expression in postnatal muscles (PSI = 4%).

In conclusion, all these patients carried biallelic TTNtv located in exons with a high PSI in fetal muscles, resulting in genotypes that appear severe and/or not to be compatible with life.

### Congenital cases improving with age

This group, including P4–9, is quite homogeneous in terms of onset (congenital) and clinical features, with all patients having hypotonia and multiple contractures at birth, but variable respiratory involvement. Importantly, the conditions of all these five patients significantly improved after birth.

Patient 4, the most severe case within this group, is an 8‐month‐old child who presented prenatally with fetal akinesia, oligohydramnios, and intrauterine growth retardation (IUGR). He required intensive care with invasive respiratory support and nasogastric feeding. He also had a syndromic‐like face with facial asymmetry with small eyes, an arched upper lip, severe micro retrognathia, and hyperconvoluted ear pinnae. He carries a homozygous TTNtv in the PEVK‐encoding exon 217, which has a PSI of 70% in fetal muscles, 20% in postnatal muscles, and is not expressed in the heart. At the last neurologic evaluation (6 months of age), he showed spontaneous breathing with stable vital parameters and improving interactive skills, even though motor skills were still severely impaired.

Patient 5 is a 4‐week‐old infant born with arthrogryposis. He carries a TTNtv in the constitutively expressed “canonical” exon 348, located in the distal A‐band, in compound heterozygosity with a TTNtv in exon 166, a metatranscript‐only exon, with a PSI of 60% in fetal muscles and 0% in postnatal skeletal and cardiac muscles. His condition was reported stable at the last examination.

Patients 6, 7, 8, and 9 are myopathic patients aged 8–33 years. They were all born with congenital hypotonia and arthrogryposis; P6 and P7 required ventilation support, and P6 additional nasogastric tube feeding. Their conditions have significantly improved with age, and now they have a milder limb‐girdle phenotype. None of them has cardiac involvement. Patients 6, 7, and 8 carry a TTNtv in an exon with high PSI in both fetal and postnatal skeletal muscles in compound heterozygosity with a TTNtv in the triplicated region of TTN (spanning from exon 173 to exon 199, encoding skeletal muscle PEVK sequences); patient 9 carries a homozygous TTNtv in the triplicated region. These exons have a low PSI in fetal skeletal muscles (15% approximately) and 0% in postnatal muscles.

To summarize, all six patients in this group have at least one variant in an exon with PSI decreasing from fetal to postnatal muscle stage.

### Congenital cases worsening with age

Patients 10, 11, and 12 showed less severe clinical phenotypes in the prenatal and perinatal period, but their signs and symptoms progressively worsened postnatally.

Patient 10 showed no contractures but diffuse hypotonia and respiratory difficulties at birth. Notably, he displayed concentric LV hypertrophy. He acquired independent walking at 20 months. At the last examination (23 months old), he was on BiPAP for respiratory failure. He carries a TTNtv in the large and constitutively expressed exon 327, in compound heterozygosity with a TTNtv in exon 13, which has a PSI of 30% in fetal muscles and of 70% in postnatal muscles. Both exons have high PSI (90% approximately) in cardiac muscles. Exon 13 is in the Z‐disk, which anchors antiparallel actin filaments from opposite sarcomere halves and forms the sarcomere boundary.^28^ This exon is included in slow and cardiac muscles but not in fast muscle. By long‐read isoform sequencing, we discovered seven novel fetal isoforms in the Z‐disk region of TTN (Fig. S2). Differential usage of exons 11–13 explains a significant part of the variation between these previously unreported fetal isoforms. In our Iso‐Seq data, exon 11 is infrequently used in fetal skeletal muscle; in all the data, it is included in only one out of 86 transcripts spanning the region. Instead, exon 13, is included in 45 out of 86 transcripts spanning this region.

Patient 11 presented with congenital arthrogryposis, hypotonia, and chest deformities; his condition deteriorated, and he died at 6 months of age of respiratory failure (see Table S1 for further details). He carried a TTNtv in the constitutively expressed exon 312, in compound heterozygosity with a TTNtv in the PEVK‐encoding exon 133 which is an I‐band differentially expressed exon with some fetal expression (PSI of 20% in fetal skeletal muscles) and a PSI of 30% approximately in postnatal muscles.

Patient 12 is an asymptomatic 14‐year‐old patient with recent onset of constant hyperCKemia (600–850) as the only sign of muscular disorder. His clinical history, including the perinatal period, is unremarkable. At ES, the only pathogenic finding was a homozygous TTNtv in exon 133.

In conclusion, all these patients have at least one TTNtv located in an exon with a very low PSI in fetal muscles but a higher PSI in postnatal muscles.

### An unlikely case of titinopathy

Patient 13 is an arthrogrypotic fetus with some unspecific syndromic‐like signs (macrocephaly >90 + p, vertebral fusion). No heart or muscles anomalies were found. Termination of pregnancy was carried out at 35 weeks. ES analysis showed biallelic TTN variants: a TTNtv in the canonical exon 289, in compound heterozygosity with a TTNtv in exon 45 encoding Ig‐domain 25, with an undetectable expression in both fetal and postnatal skeletal muscles in our RNAseq study. Exon 45 is a Novex‐1‐only exon, apparently expressed in the Novex1 isoform of cardiac muscle. According to Bang et al, the Novex‐1 transcript is expressed at low levels in postnatal skeletal muscles.^1^ We conclude that it seems unlikely that the fetus' phenotype might be caused by the identified biallelic TTNtv; however, excluding it with certainty would require detailed analysis of exon 45 usage in several fetal skeletal muscles.

## Discussion

To date, we do not yet have a proper understanding of the full range of titin isoforms. Nevertheless, in previous studies, many alternative splicing events (ASE) have been found, some of them at a very high level, suggesting the presence of a larger number of isoforms that are yet uncharacterized.^4^ In this study, we provided the first insight into the use of *TTN* exon usage in human fetal and postnatal skeletal muscles, both by short‐read and long‐read RNA‐sequencing, and we used the data to assess the clinical meaning of truncated variants in newly identified patients.^28^

PCA analysis on the samples cohort used for the RNA‐sequencing analysis is reported in Figures S3 and S4. As expected, we found a clear distinction of the studied sample groups (i.e., fetal skeletal muscle, postnatal skeletal muscle, fetal cardiac muscle, and postnatal cardiac muscle) based on titin exon usage, but no association between the genetic status of the sample (myopathic or healthy controls) and titin expression profile.

**Figure 1:**
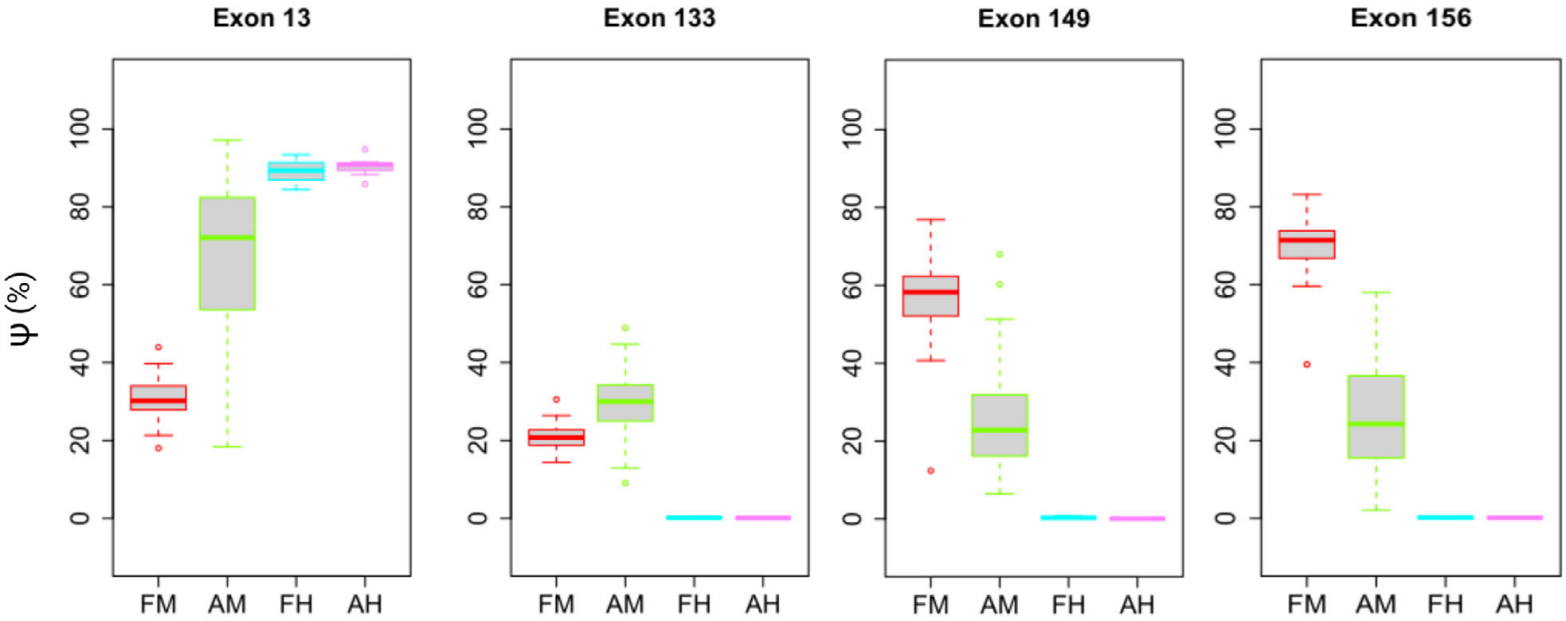
Exon usage of “false” canonical exons. Although exons 13,133, 149, and 156 are traditionally described as canonical exons included in postnatally expressed skeletal muscle isoforms, our analysis shows that all of them have a variable PSI score. AH, postnatal heart; AM, postnatal muscles; FH, fetal heart; FM, fetal muscles.

**Figure 2:**
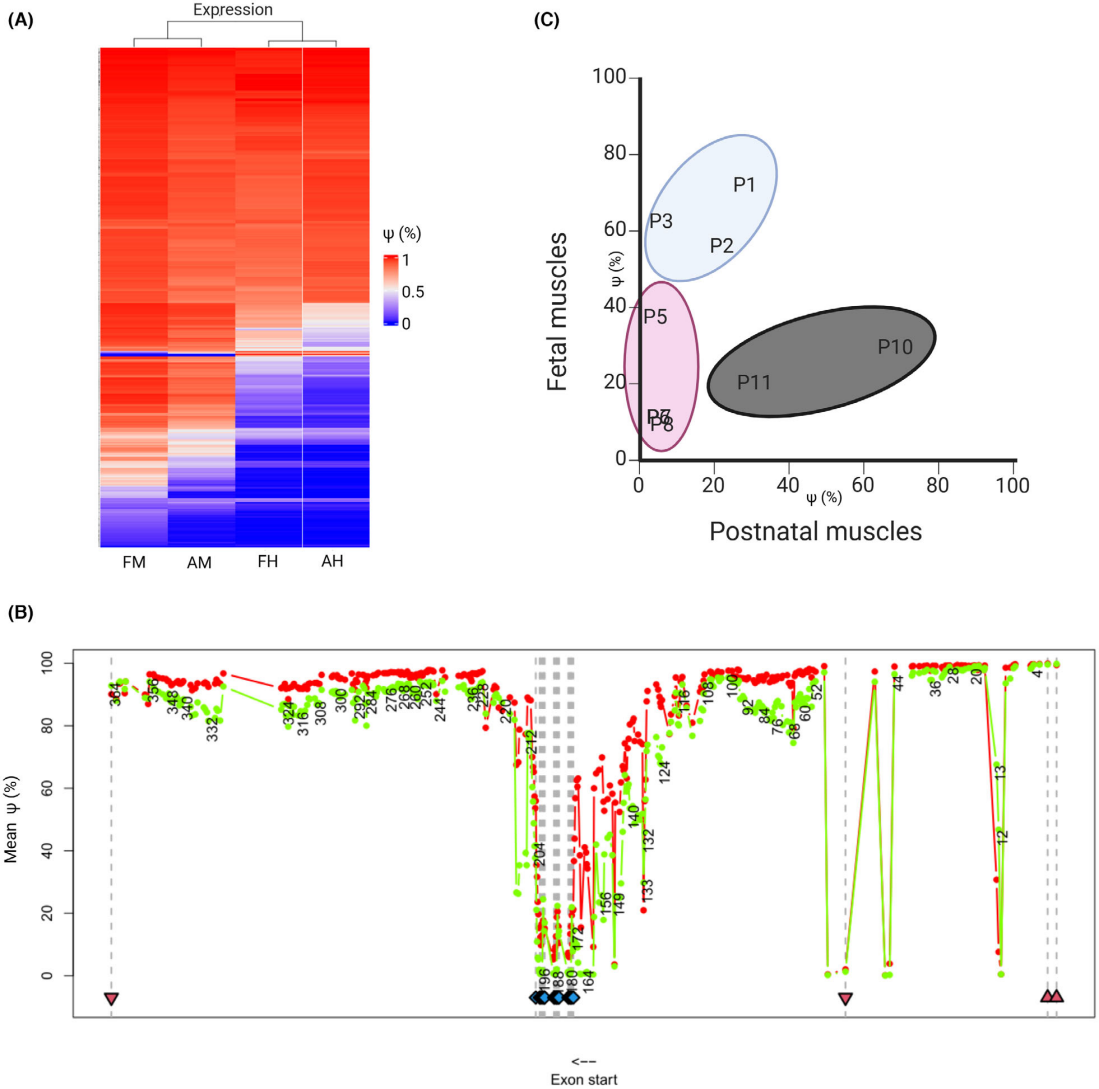
*TTN* exon usage. (A) Heat map showing the PSI index score of *TTN* exons in skeletal and cardiac muscles in prenatal and postnatal samples. (B) PSI graphical representation in prenatal (red) and postnatal (green) skeletal muscles. (C) PSI scores of the mutated exon in postnatal (x‐axis) and fetal (y‐axis) skeletal muscles correlate with the clinical phenotype and the disease course, showing the three clusters: pre‐ or perinatal death (P1, P2, and P3 in blue); improving disease course (P5–P8 in pink); and worsening disease course (P9–P11 in dark gray).

With our analysis, we demonstrated that few exons are actually “canonical,” and most show a variable pattern (Figs. 1 and 2, Table S1). We speculate that biallelic truncating variants in canonical exons would most likely not be compatible with life, as they have never to date been reported in living individuals; nevertheless, we present five individuals with biallelic TTNtv in exons previously regarded as canonical: P1–P2 with TTNtv in exons 156 and 149, respectively, P10 carrying a TTNtv in exon 13, and P11–P12 carrying a TTNtv in exon 133 (Fig. 1). Remarkably, P10 and P12 are alive at 23 months and 15 years, respectively, and P12 with only hyperCKemia as referred symptom of muscle disease.

On the other hand, we show that some exons defined as “metatranscript‐only” are also expressed in postnatal skeletal muscles, for example, exon 217 (P4). In addition, a case of a 33‐year‐old male patient with a homozygous truncating variant in the so‐called metatranscript exon 170 was published recently.^23^ Our data suggest that exon 170 has a PSI of 57% in fetal muscles and 10% in postnatal muscles, emphasizing that a new classification of *TTN* exons is needed. Moreover, we suggest that the terms canonical exons and metatranscript only exons should thereby be avoided, and each exon should be defined by its PSI in fetal or postnatal muscles.

Our study, combining short‐read RNA‐sequencing and PacBio Iso‐Seq, represents a first step toward a deeper understanding of titin isoforms.

By long‐read sequencing, we found seven different novel fetal isoforms spanning the Z‐disk (Fig. S2), which is a particularly interesting titin region, anchoring titin to the actin cytoskeleton at the sarcomere boundary. This region of titin includes the first four immunoglobulin‐type domains (Z1–Z4) and a series of 45‐residue repeats, called Z‐repeats. As expected, differential usage of exons 11, 12, and 13 (which are part of the Z‐repeats) explains a significant part of the variation between these isoforms, and that, in our data, exon 11 is very rarely used in fetal skeletal muscles. It is probable that multiple isoforms with differential exon usage of Z‐repeats create minor but significant protein‐level differences affecting titin anchoring. This is consistent with finding of different thickness and protein composition of Z‐disks across species and muscle types.^29^ Interestingly, exon 13, which hosts a TTNtv in the case of P10 (who presented with hypotonia and cardiomyopathy since birth), showed a higher inclusion level.

Regarding the complex I‐band region of titin, we know that it is composed of two principal stretches composed by tandemly arranged Ig domains, intercalated by the PEVK sequence that has no defined structure and acts as an entropic elastic spring.^2^ These exons are differentially expressed in tissue‐specific transcripts, which results in the characteristic size of the main postnatal titin isoforms.^4^ However, in our fetal samples analyzed by long‐read sequencing, we did not find any I‐band pattern of so‐called combined alternative splicing, where a certain splicing event always takes place in combination with another splicing event. This observation seems to further confirm that, at prenatal age, I‐band exons, especially from the PEVK exons, whose splice donor and acceptor sites are highly compatible, are not expressed “in blocks,” as there are still no defined postnatal isoforms. These findings are also consistent with our short‐read sequencing data indicating that some I‐band exons are more expressed in fetal than in postnatal skeletal muscles. Indeed, some of these exons are mutated in cases of patients who improve after birth, for example, P6, P7, and P8 with variants in the triplicated region, which is part of the PEVK domain.

Clearly, our study has several limitations: first of all, a thorough characterization of titin splicing pattern would benefit from a larger number of samples.

Secondly, within the triplicated region (exons 173–199), there may be technical difficulties in mapping the single exon accurately. Furthermore, the applicability of our model may not always be straightforward for all titin variants. For example, splicing variants, which were not included in this study, pose additional challenges.^30^ Further analyses will be needed to deepen our knowledge on differential exon usage: for example, according to the skeletal muscle type (fast‐twitch, slow‐twitch), body localization, stage of development (week of pregnancy), and other factors. Considering that titin is the largest and probably one of the most complex proteins in animals, we are far from being able to fully explain all the different signs and symptoms within the titinopathy spectrum. However, we show here that exon usage represents a valuable guidance to predict the prognostic trajectory of most of the biallelic titinopathy cases, with major implications for physicians and patients. As a proof, we applied retrospectively this model to other published cases of biallelic titinopathies, which were outlined in our recent publication (Di Feo et al, 2023), and found that most of the cases with clinical information on the prenatal phenotype and/or on the first years of life have a disease course fitting the exon usage trajectory (Table S4).^15^, ^18^, ^20^, ^31^, ^32^

Interestingly, our exon usage study not only provides a better understanding of biallelic titinopathies but also allows us to avoid misdiagnosis in prenatal cases. For example, by combining clinical and molecular knowledge, it will be possible to rule out that a TTNtv in an exon with a very low PSI in fetal skeletal muscles could be the cause of a severe prenatal phenotype.

Obviously, clinical predictions based on exon usage data do not consider many factors, for example, the presence of modifying variants in other genes (e.g., *SRPK3* or *RBM20*) or the coexistence of other potentially damaging *TTN* missense variants.^33^, ^34^, ^35^ In fact, in the presence of a TTNtv in an exon with a PSI below 100%, the remaining transcripts with the exon spliced out may, on the other hand, include other potentially damaging variants. Future investigations could shed new light on the importance of in‐cis variants in biallelic titinopathies and, perhaps even change a genetic paradigm, in particular that the presence of a variant in cis with a pathogenic variant in any inheritance pattern is considered a criterion of benignity (BP2) according to ACMG guidelines.^36^

Also, there may be several variables in the clinical course that we are unable to predict with exon usage data alone (including response to treatments and infections). In P2, we cannot exclude the possibility that if the fetus had not gone through TOP, this case might have improved, as the PSI of exon 149 in fetal muscles is 55% while in the postnatal period it drops by about 30%.

## Conclusions

With the advent of the genomic era, it has become crucial to understand the clinical effects of variants in large and complex genes. Not considering alternative transcripts and differential tissue expression can lead to missed or incorrect diagnoses, and this is still a major issue in many diagnostic settings.^37^ *TTN* is one of the genes that face frequent challenges in variant clinical interpretation, as TTNtv are found in approximately 1 in 100 individuals and very rare missense *TTN* variants in approximately 20 in 100.^38^, ^39^ Our study provides a framework, based on exon usage, that can be applied to other genetic diseases, as recent studies have shown that 70% of the human genes have at least 15 transcript isoforms.^40^

## Funding Information

M.S. received support from the Academy of Finland (grant 339437), Association Française contre les Myopathies (grant 23281), Sydäntutkimussäätiö, and Samfundet Folkhälsan i Svenska, Finland. A.O. received supported by Magnus Ehrnrooth Foundation. B.U. received support from the European Joint Program on Rare Diseases (project IDOLS‐G), Academy of Finland, Juselius Foundation, and Samfundet Folkhälsan i Svenska Finland. F.M. received support from the European Joint Program on Rare Diseases (project IDOLS‐G) and Instituto de Salud Carlos III, Spain (project number AC19/00048). P.H. received support from the Jane and Aatos Erkko foundation.

## Conflict of Interest

The authors have no conflicts of interests to declare.

## Author Contributions

M.F.D.F., M.S., B.U., and A.O. contributed to the conception and design of the study; M.F.D.F., M.G., H.J., F.F., E.M., C.C., I.K., D.G.A., A.F.B., M.I., A.C., L.P., D.N.D.B., A.N.O., S.A.K., T.A.B, S.N., E.R., M.M., S.G., B.C., J.S., J.T., F.M., J.C.S., M.A.S.D., and H.T. contributed to acquisition of data; M.F.D.F., A.O., and E.N., contributed to acquisition of data and data analysis; M.F.D.F., M.S., B.U., M.J., E.N., and A.O. contributed to drafting the text or preparing the figures.

## Supporting information


Data S1.

## Data Availability

The data that support the findings of this study are available from the corresponding author upon reasonable request.
