# Erratum to “Inferring Disease Course From Differential Exon Usage in the Wide Titinopathy Spectrum”

**DOI:** 10.1002/acn3.52274

**Published:** 2025-05-29

Di Feo MF, Oghabian A, Nippala E, et al. Inferring disease course from differential exon usage in the wide titinopathy spectrum. Ann Clin Transl Neurol. 2024;11(10):2745‐2755. doi: 10.1002/acn3.52189

The following ethics statement has been inadvertently omitted during the publication process: “Ethical approval for this study falls under HUS/16896/2022. Fetal sampling falls under the approval by Ethics Committee of Clinical Research of the Hospital Universitari Vall d'Hebrón (PR(AMI)210/2021).”

We apologize for this error.

