# Supplementary material for: Inferring disease course from differential exon usage in the wide titinopathy spectrum
Source: Ann Clin Transl Neurol. 2024 Aug 28;11(10):2745–55. doi: 10.1002/acn3.52189 (PMC11514934; doi:10.1002/acn3.52189)
Supplement: Supplementary file 1 — Data S1. [file ACN3-11-2745-s001.docx]

**Supplementary Material**

**Supplemental Methods**

**Figure S1.** Schematic representation of long-read and short-read RNA-sequencing methodological pipelines

**Figure S2**. Z-disk isoforms identified by Iso-Seq

**Table S1.** Patients’ clinical details

**Figure S3.** PCA analysis on the overall samples cohort

**Figure S4.** PCA analysis comparing samples from titinopathy cases and other samples in the cohort

**Table S2.** Technical info on the internal cohort of adult individuals for RNA-seq

**Table S3.** PSI data from short-read RNA-sequencing

**Table S4.** Published cases in the literature with longitudinal clinical info, fitting the expected disease course

Link to <https://gacatag.shinyapps.io/TTN_PSIVIS/> (short-read database)

**Supplemental Methods**

**Publicly available muscle/heart databases**

We used RNA-seq data on 4 samples retrieved from a publicly available database (ENCODE), with accession IDs ENCFF001RMW, ENCFF001ROG, ENCFF001RPA and ENCFF001RPE.

**RNA-sequencing on sample biopsies**

Sample biopsies were taken from an internal cohort of 41 individuals. Among them, 13 are solved cases with a diagnosis of genetic myopathy, 23 are unsolved cases with myopathic signs and symptoms, four are non-myopathic patients who have performed biopsy due to hyperCKemia, and one is an otherwise healthy patient who underwent leg amputation due to diabetic complications (eTable 3).

For short-read RNA-sequencing, we used fetal skeletal muscles (n=20) and fetal heart muscles (n=2) from 2 different fetuses, without muscle pathology, obtained from voluntary TOPs. The mothers signed the informed consent. Data was anonymized for further analysis. A trained fetal pathologist performed fetal autopsy in less than 12 hours times after fetal expulsion and dissected a sample from five different skeletal muscles and heart muscles. Tissues were frozen and stored at -80ºC.

For long-read sequencing, data was generated from five different skeletal muscles and one fetal heart muscle, belonging to the same fetus.

RNA was extracted with Qiagen RNeasy Plus Universal Mini Kit (Qiagen, Hilden, Germany) and sequencing libraries were constructed by Oxford Nanopore. The sequencing was performed on a PacBio Sequel II sequencer.

**Splicing analysis and exon inclusion level estimation**

IntEREst supports several methods for summarization of the mapped RNA-seq reads, PSI measurement, and statistical tests to compare the number of reads that map to the introns/exons in different samples. These tools are especially useful for studying RNA splicing across the studied samples.^27^ The PSI value (which is also denoted with Ψ) for exon usage is measured with the fraction of the reads that span the flanking introns (IS) to the sum of this number with twice the number of reads that skip the exon (ES) (*formula 1*). The measured PSI is a value in the range of 0 to100. If all RNAs include the exon then PSI is 100. On the contrary, if none of the exons are included in the mRNAs then PSI is zero.

$\Psi=\frac{IS}{IS+2\times ES}$ (*formula 1*)

**Identification of new isoforms**

The Iso-Seq data was processed by the company providing the sequencing (Oxford Nanopore), using the SMRT link software v. [10.0.0.108728](https://github.com/PacificBiosciences/IsoSeq_SA3nUP/wiki). Consensus calling, clustering, and classification was performed with default parameters. Additionally, the company provided transcriptome analysis and isoform characterization, performed with the SQANTI software with default parameters.^28^

SQANTI classifies transcripts based on their splice junctions and their splice donor and acceptor sites. From the classification obtained with SQANTI, only transcripts related to TTN (Ensembl ID ENSG00000155657) were included in the analysis and duplicates were filtered out. The extracted sequences were aligned and identified using the BLAST-like Alignment tool (BLAT). This allowed for identification and documentation of every exon covered by each transcript, enabling us to identify the isoforms.

**Figure S1.** Schematic representation of long-read and short-read RNA-sequencing methodological pipelines


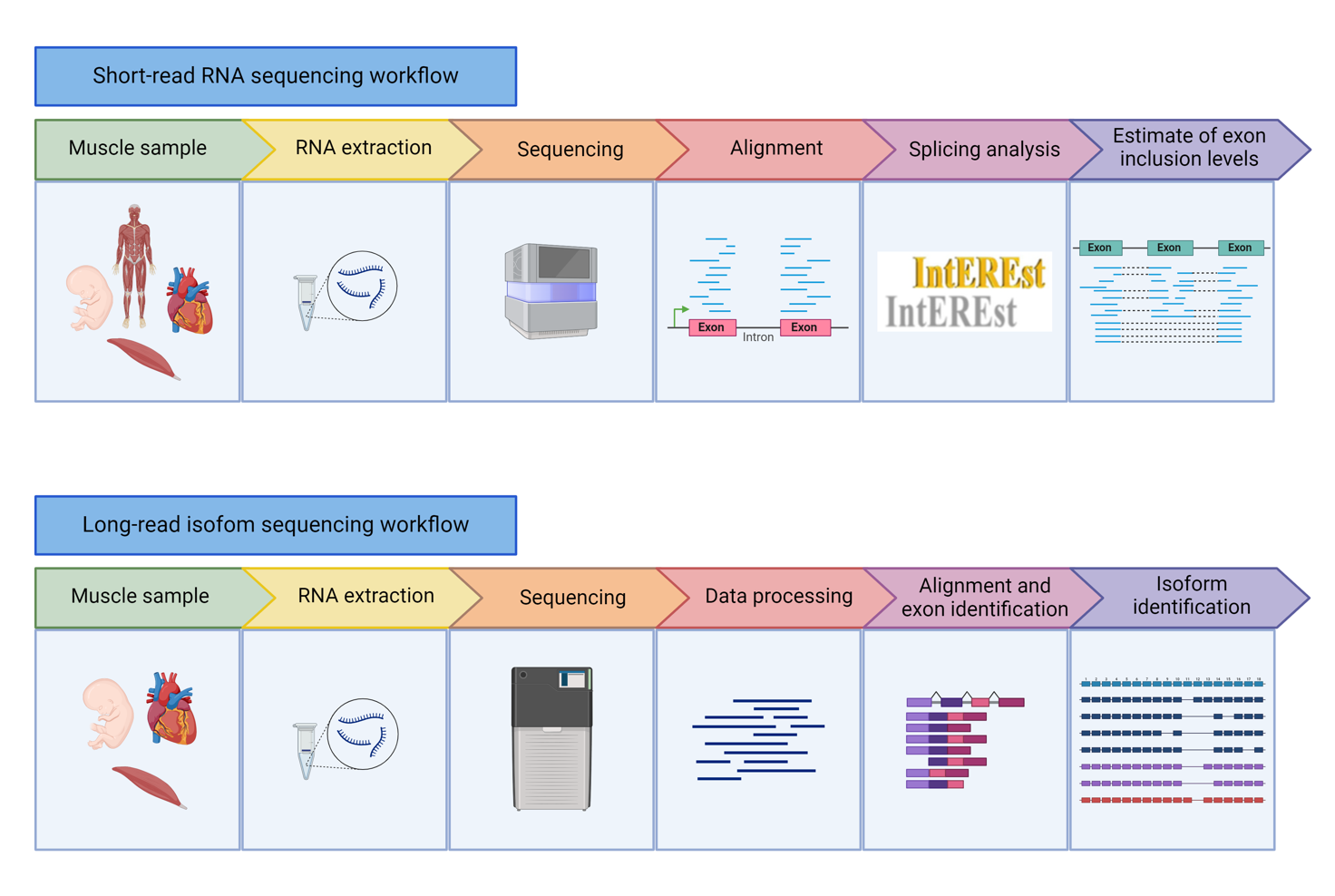


**Figure S2.** Z-disk isoforms identified by Iso-Seq


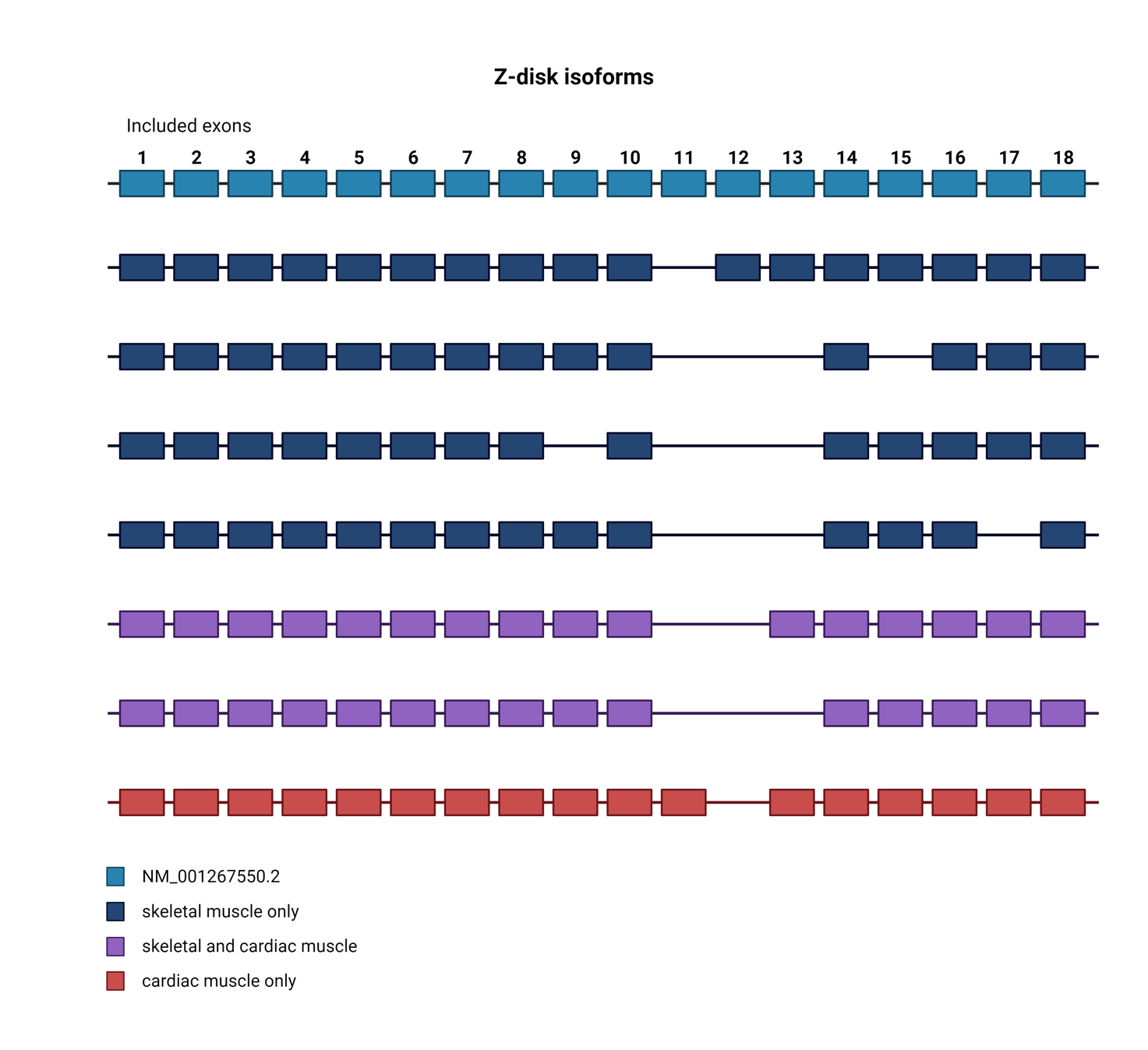


**Table S1.** Patients’ clinical details

| **Patient ID** | P1 | P2 | P3 | P4 | P5 | P6 |
| --- | --- | --- | --- | --- | --- | --- |
| **Category** | Deceased before birth | Deceased before birth | Deceased after birth | Improving with age | Improving with age | Improving with age |
| **Affected individuals in the same family** | No | No | No | 2 TOP | No | No |
| **Consanguinity** | No | No | No | Not declared | No | No |
| **Maternal disease** | No | N/A | No | No | No | No |
| **Paternal disease** | No | N/A | No | No | No | No |
| **Age interval at onset** | Prenatal | Prenatal | Prenatal | Prenatal | Prenatal | Prenatal |
| **Age interval at last examination** | Prenatal | Prenatal | Birth | 6-12 months | 0-6 months | 5-10 years |
| **Prenatal associated signs** | IUGR << 5th centile, fetal akinesia with hypo/amyoplasia, discrete hydrops | Arthrogryposis | Placental abruption | Fetal akinesia, oligohydramnios, IUGR | No | Malformations in feet and hands |
| **Fetal death** | No | No | No | No | No | No |
| **TOP (week)** | Yes, 27 weeks | Yes | No | No | No | No |
| **Delivery (week)** | N/A | N/A | 31 | 37 | 40 | 35 (cesarean) |
| **Contractures** | Yes | Yes | Yes | Yes | Yes | Yes |
| **Respiratory difficulties** | N/A | N/A | Died at 55 minutes | Yes, intubated | No | Nasal goggles only during the first day of life |
| **Feeding difficulties** | N/A | N/A | N/A | Yes, swallowing deficit and sialorrhea | Yes | Nasogastric tube feeding during the first week of life |
| **Generalized hypotonia** | N/A | N/A | N/A | Yes | Yes | Yes, at newborn period |
| **Intellectual disability** | N/A | N/A | N/A | Normal EEG | N/A | No |
| **Cardiac abnormalities** | N/A | N/A | No | No | No | No |
| **Dysmorphic features** | N/A | N/A | Hypertelorism, upturned nose, short philtrum, tent-shaped mouth, low set ears, micrognathia | Facial asymmetry, small eyes, arched upper lip, severe micro-retrognathia, hyper-convoluted ear pinnae | No | Facial dysmorphisms compatible with myopathic face |
| **Other signs detected after birth  (at autopsy or at clinical examination)** | N/A | N/A | Yes, abnormal palmar creases and rocker bottom feet. He had small scrotum, evidence of hydrops (facial and neck oedema at delivery, fluid in the pericardial and plural cavities) | Multiple congenital fractures | Floppy infant, areflexia, movement disorder | Fracture of both humeri during childbirth |
| **Postnatal death** | N/A | N/A | Yes, 1 hour after birth | No | No | No |
| **Other postnatal associated signs** | N/A | N/A | N/A | Not reported | No | Facial, proximal and distal weakness, scoliosis |
| **Improved after birth** | N/A | N/A | N/A | Yes | Yes | Yes |
| **Independent walking** | N/A | N/A | N/A | N/A | N/A | No |
| **Var 1** | c.14183dup, (p.Asn4728LysfsTer8) | c.23386C>T, (p.Arg7796Ter) | c.85348A>T, (p.Lys28450Ter) | c.40267G>T,  (p.Glu13423Ter) | c.96464del, (p.Val32155Glu  fsTer16) | c.33055del, (p.Glu11019Ser  fsTer21) |
| **Exon var 1** | 50 | 82 | 327 | 217 | 348 | 136 |
| **Exon usage fetal muscle** | 99% | 95% | 97% | 68% | 94% | 77% |
| **Exon usage adult muscle** | 97% | 86% | 93% | 27% | 90% | 51% |
| **Exon usage fetal heart** | 100% | 64% | 93% | 1% | 85% | 42% |
| **Exon usage adult heart** | 98% | 40% | 95% | 0% | 91% | 27% |
| **Cardiodb** |  |  |  |  |  |  |
| **Var 2** | c.35182_35188del (p.Ala11728LysfsTer50) | c.34408del, (p.Lys11470GlufsTer20) | c.36100_36101del, (p.Glu12034ThrfsTer5) | c.40267G>T,  (p.Glu13423Ter) | c.36040A>T, (p.Lys12014*) | c.38737G>T, (p.Glu12913Ter) |
| **Exon var 2** | 156 | 149 | 167 | 217 | 166 | 199 |
| **Exon usage fetal muscle** | 70% | 55% | 63% | 68% | 39% | 15% |
| **Exon usage adult muscle** | 26% | 25% | 4% | 27% | 1% | 2% |
| **Exon usage fetal heart** | 0% | 0% | 1% | 1% | 0% | 2% |
| **Exon usage adult heart** | 0% | 0% | 0% | 0% | 0% | 1% |
| **Cardiodb** |  |  |  |  |  |  |
| **Hom/comp het** | Comp het | Comp het | Comp het | Hom | Comp het | Comp het |
| **Publication** | Unpublished | Laquerriere A, Et Al. 2020 | Unpublished | Unpublished | Unpublished | Unpublished |

| **Patient ID** | **P7** | **P8** | **P9** | **P10** | **P11** | **P12** | **P13** |
| --- | --- | --- | --- | --- | --- | --- | --- |
| **Category** | Improving with age | Improving with age | Improving with age | Worsening with age | Worsening with age | Worsening with age | Unlikely titinopathy |
| **Affected individuals in the same family** | No | No | No | No | No | No | No |
| **Consanguinity** | No | No | No | No | No | Yes | No |
| **Maternal disease** | No | No | No | No | N/A | No | No |
| **Paternal disease** | No | No | No | No | N/A | No | No |
| **Age interval at onset** | Prenatal | Prenatal | Prenatal | 20-24 months | Prenatal | 10-15 years | Prenatal |
| **Age interval at last examination** | 15-20 years | 35-40 years | 10-15 years | 2-4 years | Prenatal | 15-20 years | Prenatal |
| **Prenatal associated signs** | Unknown | Unknown | No | N/A | Threatened miscarriage | No | Macrocephaly > 90+p, vertebral fusion D7-D8-D9, cystic lymphangioma of the mesentery |
| **Fetal death** | No | No | No | No | No | No | No |
| **TOP (week)** | No | No | No | No | No | No | Yes, 35 |
| **Delivery (week)** | Unknown | Unknown | 38 | Caesarean at term | 38 week | N/A | N/A |
| **Contractures** | Yes | Yes | Yes | No | Yes | No | Yes |
| **Respiratory difficulties** | Reduced vital capacity | No | Yes, pneumonia and extensive atelectasis at 14 months | Yes | Yes | No | N/A |
| **Feeding difficulties** | No | No | Yes | No | Yes | No | N/A |
| **Generalized hypotonia** | Yes, at newborn period | Yes, at newborn period | Yes | Yes | Yes | No | No |
| **Intellectual disability** | No | No | No | No | Yes (post-hypoxic changes) | No | N/A |
| **Cardiac abnormalities** | No | No | No | Yes, concentric LV hypertrophy | No | No | N/A |
| **Dysmorphic features** | No | No | No | No | No | No | N/A |
| **Other signs detected after birth  (at autopsy or at clinical examination)** | No | No | Both feet in valgus with adducted metatarsus; deviated hands, with contractures of the finger flexors. Sharp fingers. | Hypotonia, sluggish reflexes | Unfused arches C5-T2, T12, dysembryogenesis of spinal column and chest | No | N/A |
| **Postnatal death** | No | No | No | No | Yes, 6 months | No | N/A |
| **Other postnatal associated signs** | Proximal and distal weakness | Proximal and distal weakness | No | No | Not reported | HyperCKemia (600-850) | N/A |
| **Improved after birth** | Yes | Yes | Yes | No | N/A | No | N/A |
| **Independent walking** | Yes | With aids | Never | 23 months | N/A | Yes | N/A |
| **Var 1** | c.32656C>T, (p.Gln10886Ter) | c.103531A>T, (p.Lys34511Ter) | c.38661_38665del, p.(Lys12887AsnfsTer6) | c.70978C>T, (p.Arg23660Ter) | c.65163T>A, (p.Cys21721Ter) | c.32656C>T, (p.Gln10886Ter) | c.55939G>T, (p.Glu18647Ter) |
| **Exon var 1** | 320 | 359 | 198 | 327 | 312 | 133 | 289 |
| **Exon usage fetal muscle** | 92% | 94% | 11% | 97% | 92% | 21% | 96% |
| **Exon usage adult muscle** | 84% | 94% | 1% | 93% | 87% | 30% | 93% |
| **Exon usage fetal heart** | 82% | 88% | 0% | 93% | 79% | 0% | 88% |
| **Exon usage adult heart** | 90% | 95% | 1% | 95% | 91% | 0% | 95% |
| **Cardiodb** | 100% | 100% | 7% | 100% | 100% | 10% | 100% |
| **Var 2** | c.38737G>T, (p.Glu12913  Ter) | c.38661_38665del, (p.Lys12887Asn  fsTer6) | c.38661_38665del, (p.Lys12887Asn  fsTer6) | c.2047C>T, (p.Gln683Ter) | c.32680del, (p.Val10895LeufsTer3) | c.32656C>T, (p.Gln10886  Ter) | c.10439dupA, (p.Thr3481Asp  fsTer21) |
| **Exon var 2** | 199 | 198 | 198 | 13 | 133 | 133 | 45 |
| **Exon usage fetal muscle** | 15% | 11% | 11% | 31% | 21% | 21% | 4% |
| **Exon usage adult muscle** | 2% | 1% | 1% | 68% | 30% | 30% | 0% |
| **Exon usage fetal heart** | 2% | 0% | 0% | 89% | 0% | 0% | 1% |
| **Exon usage adult heart** | 1% | 1% | 1% | 90% | 0% | 0% | 0% |
| **Cardiodb** | 3% | 7% | 7% | 96% | 10% | 10% | 1% |
| **Hom/comp het** | Comp het | Comp het | Hom | Comp het | Comp het | Hom | Comp het |
| **Publication** | Unpublished | Unpublished | Unpublished | Unpublished | Unpublished | Unpublished | Unpublished |

**Figure S3.** PCA analysis on the overall samples cohort

**Figure S4.** PCA analysis comparing samples from titinopathy cases and other samples in the cohort

**Table S2.** Technical info on the internal cohort of adult individuals for RNA-seq

| **Sex** | **No. samples** |
| --- | --- |
| M | 20 |
| F | 12 |
| NA | 9 |
| **Age at biopsy** |  |
| 0-4 | 4 |
| 5-10 | 2 |
| 11-20 | 4 |
| 21-60 | 23 |
| >60 | 4 |
| NA | 2 |
| **Clinical conditions** |  |
| Unsolved myopathy with FINmaj | 5 |
| Unsolved myopathy with a single TTNtv | 9 |
| Unsolved myopathy (without TTNtv) | 9 |
| Biallelic titinopathies | 11 |
| BMD (DMD deletion) | 1 |
| Digenic TTN-SRKP3 | 1 |
| Amputees for myopathy-unrelated reasons | 4 |
| Hyperckemia without muscle alterations | 1 |

**Table S3.** PSI data from short-read RNA-sequencing

| **Exon** | **Coordinates (hg38)** | **Fetal skeletal muscles** | **Adult skeletal muscles** | **Fetal cardiac muscles** | **Adult cardiac muscles** |
| --- | --- | --- | --- | --- | --- |
| **1** | chr2:178807212-178807423 | 0,994 | 0,998 | 0,987 | 0,997 |
| **2** | chr2:178804552-178804655 | 0,999 | 0,996 | 0,980 | 0,999 |
| **3** | chr2:178802138-178802341 | 0,998 | 0,996 | 0,975 | 0,998 |
| **4** | chr2:178800395-178800682 | 0,997 | 0,995 | 0,982 | 0,997 |
| **5** | chr2:178799825-178799910 | 0,996 | 0,987 | 0,980 | 0,995 |
| **6** | chr2:178799487-178799731 | 0,992 | 0,982 | 0,978 | 0,994 |
| **7** | chr2:178794922-178795252 | 0,997 | 0,990 | 0,972 | 0,998 |
| **8** | chr2:178794399-178794551 | 0,995 | 0,984 | 0,971 | 0,997 |
| **9** | chr2:178793404-178793541 | 0,987 | 0,951 | 0,969 | 0,995 |
| **10** | chr2:178792072-178792197 | 0,986 | 0,936 | 0,973 | 0,988 |
| **11** | chr2:178790708-178790845 | 0,004 | 0,004 | 0,712 | 0,559 |
| **12** | chr2:178789978-178790115 | 0,076 | 0,467 | 0,682 | 0,618 |
| **13** | chr2:178789360-178789497 | 0,307 | 0,676 | 0,890 | 0,904 |
| **14** | chr2:178785848-178786141 | 0,993 | 0,986 | 0,983 | 0,996 |
| **15** | chr2:178785620-178785742 | 0,978 | 0,979 | 0,969 | 0,983 |
| **16** | chr2:178784070-178784351 | 0,994 | 0,992 | 0,979 | 0,995 |
| **17** | chr2:178783720-178783785 | 0,979 | 0,983 | 0,974 | 0,987 |
| **18** | chr2:178782806-178783064 | 0,994 | 0,991 | 0,974 | 0,995 |
| **19** | chr2:178782539-178782602 | 0,988 | 0,977 | 0,969 | 0,989 |
| **20** | chr2:178782212-178782427 | 0,992 | 0,985 | 0,976 | 0,995 |
| **21** | chr2:178781121-178781263 | 0,992 | 0,985 | 0,976 | 0,993 |
| **22** | chr2:178780000-178780205 | 0,993 | 0,981 | 0,982 | 0,992 |
| **23** | chr2:178779229-178779462 | 0,993 | 0,977 | 0,976 | 0,991 |
| **24** | chr2:178778874-178779118 | 0,994 | 0,978 | 0,985 | 0,993 |
| **25** | chr2:178777704-178777975 | 0,995 | 0,980 | 0,984 | 0,992 |
| **26** | chr2:178777420-178777584 | 0,992 | 0,978 | 0,980 | 0,989 |
| **27** | chr2:178777149-178777317 | 0,991 | 0,978 | 0,962 | 0,992 |
| **28** | chr2:178775356-178777049 | 0,993 | 0,989 | 0,995 | 0,995 |
| **29** | chr2:178774921-178775202 | 0,993 | 0,980 | 0,995 | 0,991 |
| **30** | chr2:178774207-178774473 | 0,993 | 0,976 | 0,999 | 0,989 |
| **31** | chr2:178773838-178774110 | 0,984 | 0,967 | 0,966 | 0,983 |
| **32** | chr2:178773462-178773725 | 0,982 | 0,962 | 0,957 | 0,984 |
| **33** | chr2:178773109-178773369 | 0,988 | 0,955 | 0,999 | 0,989 |
| **34** | chr2:178771211-178771471 | 0,992 | 0,973 | 0,999 | 0,992 |
| **35** | chr2:178770412-178770675 | 0,992 | 0,976 | 0,999 | 0,993 |
| **36** | chr2:178770060-178770320 | 0,983 | 0,946 | 0,998 | 0,991 |
| **37** | chr2:178769679-178769939 | 0,976 | 0,946 | 0,988 | 0,978 |
| **38** | chr2:178768673-178768933 | 0,993 | 0,979 | 0,999 | 0,991 |
| **39** | chr2:178768014-178768155 | 0,992 | 0,974 | 0,999 | 0,993 |
| **40** | chr2:178767759-178767924 | 0,991 | 0,972 | 0,999 | 0,992 |
| **41** | chr2:178766381-178766612 | 0,991 | 0,969 | 0,999 | 0,994 |
| **42** | chr2:178764527-178764811 | 0,992 | 0,971 | 0,997 | 0,994 |
| **43** | chr2:178764177-178764302 | 0,990 | 0,961 | 0,997 | 0,992 |
| **44** | chr2:178758984-178759172 | 0,990 | 0,965 | 0,999 | 0,993 |
| **45** | chr2:178757542-178757916 | 0,038 | 0,003 | 0,006 | 0,005 |
| **46** | chr2:178756222-178756797 | 0,001 | 0,001 | 0,002 | 0,002 |
| **47** | chr2:178753124-178753180 | 0,974 | 0,941 | 0,989 | 0,969 |
| **48** | chr2:178744405-178752039 | 0,020 | 0,013 | 0,026 | 0,035 |
| **49** | chr2:178739141-178741921 | 0,004 | 0,002 | 0,990 | 0,979 |
| **50** | chr2:178738082-178738360 | 0,991 | 0,971 | 0,995 | 0,984 |
| **51** | chr2:178735511-178736074 | 0,973 | 0,942 | 0,376 | 0,138 |
| **52** | chr2:178734707-178734988 | 0,979 | 0,936 | 0,151 | 0,033 |
| **53** | chr2:178734328-178734606 | 0,955 | 0,861 | 0,127 | 0,031 |
| **54** | chr2:178733614-178733892 | 0,982 | 0,917 | 0,167 | 0,043 |
| **55** | chr2:178733239-178733517 | 0,972 | 0,887 | 0,156 | 0,040 |
| **56** | chr2:178732834-178733121 | 0,977 | 0,889 | 0,179 | 0,035 |
| **57** | chr2:178732440-178732718 | 0,971 | 0,867 | 0,161 | 0,033 |
| **58** | chr2:178732066-178732347 | 0,972 | 0,887 | 0,183 | 0,045 |
| **59** | chr2:178731693-178731971 | 0,964 | 0,876 | 0,181 | 0,042 |
| **60** | chr2:178731305-178731583 | 0,966 | 0,868 | 0,248 | 0,073 |
| **61** | chr2:178730925-178731203 | 0,975 | 0,901 | 0,261 | 0,086 |
| **62** | chr2:178730505-178730792 | 0,974 | 0,891 | 0,265 | 0,077 |
| **63** | chr2:178730093-178730371 | 0,969 | 0,881 | 0,242 | 0,068 |
| **64** | chr2:178729664-178729945 | 0,969 | 0,860 | 0,233 | 0,068 |
| **65** | chr2:178729288-178729566 | 0,959 | 0,874 | 0,251 | 0,080 |
| **66** | chr2:178728891-178729169 | 0,793 | 0,745 | 0,173 | 0,042 |
| **67** | chr2:178728500-178728778 | 0,938 | 0,802 | 0,178 | 0,049 |
| **68** | chr2:178728110-178728397 | 0,928 | 0,811 | 0,151 | 0,041 |
| **69** | chr2:178727585-178727863 | 0,937 | 0,779 | 0,205 | 0,065 |
| **70** | chr2:178727090-178727371 | 0,962 | 0,814 | 0,471 | 0,211 |
| **71** | chr2:178725768-178726046 | 0,960 | 0,803 | 0,478 | 0,217 |
| **72** | chr2:178725368-178725649 | 0,953 | 0,863 | 0,405 | 0,160 |
| **73** | chr2:178724260-178724538 | 0,958 | 0,880 | 0,385 | 0,168 |
| **74** | chr2:178723856-178724143 | 0,947 | 0,874 | 0,383 | 0,157 |
| **75** | chr2:178723418-178723696 | 0,955 | 0,860 | 0,412 | 0,185 |
| **76** | chr2:178723046-178723324 | 0,944 | 0,825 | 0,382 | 0,162 |
| **77** | chr2:178722659-178722937 | 0,955 | 0,842 | 0,387 | 0,179 |
| **78** | chr2:178722259-178722546 | 0,943 | 0,816 | 0,337 | 0,132 |
| **79** | chr2:178721847-178722134 | 0,940 | 0,823 | 0,409 | 0,209 |
| **80** | chr2:178720921-178721202 | 0,963 | 0,850 | 0,645 | 0,371 |
| **81** | chr2:178720385-178720663 | 0,957 | 0,842 | 0,650 | 0,390 |
| **82** | chr2:178719983-178720264 | 0,945 | 0,857 | 0,644 | 0,403 |
| **83** | chr2:178719554-178719832 | 0,958 | 0,864 | 0,641 | 0,425 |
| **84** | chr2:178719164-178719451 | 0,955 | 0,861 | 0,311 | 0,069 |
| **85** | chr2:178718695-178718973 | 0,957 | 0,875 | 0,234 | 0,041 |
| **86** | chr2:178718322-178718600 | 0,943 | 0,862 | 0,192 | 0,034 |
| **87** | chr2:178717943-178718221 | 0,944 | 0,857 | 0,219 | 0,033 |
| **88** | chr2:178717523-178717810 | 0,937 | 0,842 | 0,233 | 0,031 |
| **89** | chr2:178717095-178717382 | 0,945 | 0,850 | 0,287 | 0,056 |
| **90** | chr2:178715493-178715774 | 0,954 | 0,853 | 0,649 | 0,452 |
| **91** | chr2:178714986-178715264 | 0,948 | 0,851 | 0,705 | 0,501 |
| **92** | chr2:178714292-178714573 | 0,951 | 0,887 | 0,713 | 0,533 |
| **93** | chr2:178713897-178714175 | 0,952 | 0,895 | 0,745 | 0,556 |
| **94** | chr2:178713085-178713372 | 0,953 | 0,886 | 0,740 | 0,579 |
| **95** | chr2:178712697-178712975 | 0,978 | 0,944 | 0,790 | 0,567 |
| **96** | chr2:178712315-178712593 | 0,980 | 0,935 | 0,807 | 0,565 |
| **97** | chr2:178711944-178712222 | 0,958 | 0,938 | 0,706 | 0,547 |
| **98** | chr2:178711062-178711349 | 0,959 | 0,939 | 0,700 | 0,512 |
| **99** | chr2:178710635-178710922 | 0,976 | 0,944 | 0,774 | 0,570 |
| **100** | chr2:178709566-178709856 | 0,977 | 0,954 | 0,769 | 0,594 |
| **101** | chr2:178707526-178707813 | 0,971 | 0,952 | 0,736 | 0,583 |
| **102** | chr2:178706862-178706954 | 0,969 | 0,943 | 0,694 | 0,538 |
| **103** | chr2:178706454-178706739 | 0,974 | 0,954 | 0,762 | 0,571 |
| **104** | chr2:178705174-178705357 | 0,962 | 0,925 | 0,726 | 0,470 |
| **105** | chr2:178704877-178704966 | 0,959 | 0,929 | 0,688 | 0,488 |
| **106** | chr2:178704510-178704777 | 0,972 | 0,947 | 0,744 | 0,560 |
| **107** | chr2:178704147-178704407 | 0,973 | 0,949 | 0,724 | 0,566 |
| **108** | chr2:178702454-178702663 | 0,964 | 0,931 | 0,695 | 0,509 |
| **109** | chr2:178702168-178702245 | 0,944 | 0,903 | 0,610 | 0,367 |
| **110** | chr2:178702040-178702066 | 0,923 | 0,850 | 0,562 | 0,297 |
| **111** | chr2:178701528-178701587 | 0,924 | 0,855 | 0,589 | 0,298 |
| **112** | chr2:178701120-178701203 | 0,909 | 0,816 | 0,567 | 0,276 |
| **113** | chr2:178698843-178698914 | 0,809 | 0,768 | 0,466 | 0,243 |
| **114** | chr2:178697121-178697168 | 0,865 | 0,858 | 0,480 | 0,347 |
| **115** | chr2:178695865-178696269 | 0,956 | 0,932 | 0,599 | 0,428 |
| **116** | chr2:178695348-178695410 | 0,942 | 0,904 | 0,540 | 0,403 |
| **117** | chr2:178694829-178694906 | 0,833 | 0,801 | 0,488 | 0,466 |
| **118** | chr2:178694599-178694676 | 0,869 | 0,805 | 0,501 | 0,502 |
| **119** | chr2:178693922-178694008 | 0,955 | 0,906 | 0,369 | 0,449 |
| **120** | chr2:178693609-178693689 | 0,896 | 0,849 | 0,219 | 0,336 |
| **121** | chr2:178692497-178692580 | 0,837 | 0,813 | 0,214 | 0,213 |
| **122** | chr2:178692016-178692099 | 0,774 | 0,776 | 0,176 | 0,135 |
| **123** | chr2:178689813-178689896 | 0,882 | 0,731 | 0,389 | 0,284 |
| **124** | chr2:178689515-178689595 | 0,861 | 0,678 | 0,047 | 0,039 |
| **125** | chr2:178689290-178689373 | 0,873 | 0,681 | 0,053 | 0,046 |
| **126** | chr2:178689053-178689136 | 0,893 | 0,698 | 0,051 | 0,044 |
| **127** | chr2:178688677-178688778 | 0,916 | 0,704 | 0,028 | 0,023 |
| **128** | chr2:178688111-178688224 | 0,932 | 0,764 | 0,112 | 0,065 |
| **129** | chr2:178685518-178685598 | 0,911 | 0,739 | 0,127 | 0,089 |
| **130** | chr2:178685253-178685330 | 0,878 | 0,719 | 0,074 | 0,047 |
| **131** | chr2:178684906-178684989 | 0,628 | 0,564 | 0,005 | 0,003 |
| **132** | chr2:178684666-178684749 | 0,559 | 0,459 | 0,003 | 0,001 |
| **133** | chr2:178684330-178684413 | 0,209 | 0,298 | 0,002 | 0,001 |
| **134** | chr2:178683999-178684082 | 0,741 | 0,518 | 0,031 | 0,023 |
| **135** | chr2:178683211-178683291 | 0,749 | 0,499 | 0,121 | 0,089 |
| **136** | chr2:178682697-178682903 | 0,772 | 0,514 | 0,417 | 0,272 |
| **137** | chr2:178681661-178681738 | 0,751 | 0,487 | 0,338 | 0,254 |
| **138** | chr2:178681376-178681450 | 0,823 | 0,536 | 0,005 | 0,004 |
| **139** | chr2:178681079-178681171 | 0,813 | 0,543 | 0,008 | 0,002 |
| **140** | chr2:178680254-178680331 | 0,804 | 0,536 | 0,005 | 0,001 |
| **141** | chr2:178679894-178680055 | 0,758 | 0,613 | 0,008 | 0,001 |
| **142** | chr2:178679599-178679682 | 0,728 | 0,601 | 0,007 | 0,002 |
| **143** | chr2:178679339-178679416 | 0,632 | 0,590 | 0,004 | 0,003 |
| **144** | chr2:178678747-178678830 | 0,744 | 0,642 | 0,343 | 0,304 |
| **145** | chr2:178678414-178678497 | 0,657 | 0,553 | 0,342 | 0,311 |
| **146** | chr2:178678125-178678208 | 0,671 | 0,461 | 0,375 | 0,313 |
| **147** | chr2:178677621-178677917 | 0,619 | 0,295 | 0,004 | 0,001 |
| **148** | chr2:178677201-178677287 | 0,524 | 0,250 | 0,008 | 0,004 |
| **149** | chr2:178675921-178675995 | 0,554 | 0,254 | 0,005 | 0,001 |
| **150** | chr2:178675671-178675754 | 0,036 | 0,030 | 0,003 | 0,001 |
| **151** | chr2:178675039-178675113 | 0,582 | 0,386 | 0,130 | 0,065 |
| **152** | chr2:178674314-178674409 | 0,609 | 0,451 | 0,347 | 0,246 |
| **153** | chr2:178673633-178673710 | 0,565 | 0,441 | 0,315 | 0,217 |
| **154** | chr2:178672635-178672703 | 0,528 | 0,388 | 0,072 | 0,049 |
| **155** | chr2:178672407-178672481 | 0,557 | 0,179 | 0,005 | 0,000 |
| **156** | chr2:178671971-178672267 | 0,699 | 0,257 | 0,002 | 0,001 |
| **157** | chr2:178671090-178671170 | 0,659 | 0,234 | 0,010 | 0,003 |
| **158** | chr2:178670218-178670295 | 0,648 | 0,420 | 0,256 | 0,190 |
| **159** | chr2:178669592-178669675 | 0,600 | 0,188 | 0,006 | 0,002 |
| **160** | chr2:178669373-178669447 | 0,092 | 0,004 | 0,005 | 0,000 |
| **161** | chr2:178667638-178667721 | 0,343 | 0,008 | 0,001 | 0,001 |
| **162** | chr2:178667442-178667525 | 0,357 | 0,011 | 0,003 | 0,001 |
| **163** | chr2:178667236-178667319 | 0,394 | 0,009 | 0,005 | 0,001 |
| **164** | chr2:178666824-178666901 | 0,411 | 0,007 | 0,005 | 0,001 |
| **165** | chr2:178665708-178665791 | 0,154 | 0,004 | 0,002 | 0,000 |
| **166** | chr2:178665377-178665460 | 0,385 | 0,006 | 0,002 | 0,000 |
| **167** | chr2:178664852-178664926 | 0,631 | 0,042 | 0,007 | 0,001 |
| **168** | chr2:178664654-178664737 | 0,605 | 0,090 | 0,006 | 0,001 |
| **169** | chr2:178664460-178664537 | 0,626 | 0,100 | 0,001 | 0,000 |
| **170** | chr2:178664015-178664098 | 0,568 | 0,101 | 0,006 | 0,002 |
| **171** | chr2:178663819-178663902 | 0,439 | 0,111 | 0,002 | 0,006 |
| **172** | chr2:178663627-178663710 | 0,367 | 0,145 | 0,001 | 0,000 |
| **173** | chr2:178663433-178663516 | 0,212 | 0,091 | 0,013 | 0,010 |
| **174** | chr2:178663266-178663349 | 0,209 | 0,078 | 0,022 | 0,026 |
| **175** | chr2:178662966-178663055 | 0,193 | 0,219 | 0,219 | 0,157 |
| **176** | chr2:178662729-178662812 | 0,158 | 0,015 | 0,003 | 0,002 |
| **177** | chr2:178662533-178662616 | 0,134 | 0,007 | 0,002 | 0,002 |
| **178** | chr2:178662338-178662418 | 0,068 | 0,006 | 0,000 | 0,003 |
| **179** | chr2:178662142-178662222 | 0,060 | 0,003 | 0,001 | 0,001 |
| **180** | chr2:178661948-178662028 | 0,073 | 0,007 | 0,003 | 0,003 |
| **181** | chr2:178661759-178661842 | 0,067 | 0,011 | 0,002 | 0,002 |
| **182** | chr2:178659172-178659255 | 0,105 | 0,143 | 0,032 | 0,019 |
| **183** | chr2:178659005-178659088 | 0,157 | 0,128 | 0,035 | 0,036 |
| **184** | chr2:178658705-178658794 | 0,205 | 0,223 | 0,247 | 0,182 |
| **185** | chr2:178658468-178658551 | 0,187 | 0,020 | 0,004 | 0,007 |
| **186** | chr2:178658272-178658355 | 0,126 | 0,008 | 0,002 | 0,004 |
| **187** | chr2:178658077-178658157 | 0,091 | 0,009 | 0,001 | 0,003 |
| **188** | chr2:178657881-178657961 | 0,081 | 0,004 | 0,001 | 0,001 |
| **189** | chr2:178657687-178657767 | 0,062 | 0,006 | 0,002 | 0,004 |
| **190** | chr2:178657498-178657581 | 0,053 | 0,007 | 0,004 | 0,001 |
| **191** | chr2:178654912-178654995 | 0,151 | 0,169 | 0,049 | 0,031 |
| **192** | chr2:178654745-178654828 | 0,144 | 0,143 | 0,044 | 0,043 |
| **193** | chr2:178654445-178654534 | 0,177 | 0,245 | 0,274 | 0,205 |
| **194** | chr2:178654208-178654291 | 0,130 | 0,017 | 0,003 | 0,003 |
| **195** | chr2:178654012-178654095 | 0,165 | 0,010 | 0,002 | 0,006 |
| **196** | chr2:178653817-178653897 | 0,098 | 0,012 | 0,002 | 0,004 |
| **197** | chr2:178653621-178653701 | 0,123 | 0,005 | 0,001 | 0,002 |
| **198** | chr2:178653427-178653507 | 0,110 | 0,009 | 0,003 | 0,008 |
| **199** | chr2:178653238-178653321 | 0,153 | 0,019 | 0,016 | 0,006 |
| **200** | chr2:178653041-178653124 | 0,197 | 0,052 | 0,008 | 0,005 |
| **201** | chr2:178652848-178652931 | 0,236 | 0,058 | 0,002 | 0,002 |
| **202** | chr2:178652653-178652736 | 0,316 | 0,109 | 0,003 | 0,002 |
| **203** | chr2:178652458-178652541 | 0,354 | 0,211 | 0,022 | 0,007 |
| **204** | chr2:178652264-178652347 | 0,560 | 0,417 | 0,096 | 0,065 |
| **205** | chr2:178652096-178652179 | 0,536 | 0,377 | 0,117 | 0,113 |
| **206** | chr2:178651884-178651967 | 0,574 | 0,415 | 0,105 | 0,088 |
| **207** | chr2:178651666-178651749 | 0,652 | 0,488 | 0,132 | 0,105 |
| **208** | chr2:178651453-178651536 | 0,668 | 0,558 | 0,143 | 0,110 |
| **209** | chr2:178651243-178651320 | 0,700 | 0,604 | 0,153 | 0,115 |
| **210** | chr2:178650751-178650834 | 0,882 | 0,777 | 0,171 | 0,121 |
| **211** | chr2:178650164-178650271 | 0,884 | 0,759 | 0,056 | 0,039 |
| **212** | chr2:178649817-178649894 | 0,891 | 0,776 | 0,059 | 0,046 |
| **213** | chr2:178649554-178649631 | 0,774 | 0,354 | 0,015 | 0,004 |
| **214** | chr2:178649248-178649331 | 0,773 | 0,393 | 0,007 | 0,004 |
| **215** | chr2:178647381-178647464 | 0,788 | 0,353 | 0,005 | 0,001 |
| **216** | chr2:178647064-178647144 | 0,683 | 0,262 | 0,009 | 0,006 |
| **217** | chr2:178646485-178646559 | 0,675 | 0,266 | 0,007 | 0,000 |
| **218** | chr2:178645920-178646030 | 0,874 | 0,819 | 0,031 | 0,018 |
| **219** | chr2:178644548-178644616 | 0,891 | 0,838 | 0,034 | 0,024 |
| **220** | chr2:178642237-178642317 | 0,849 | 0,875 | 0,857 | 0,904 |
| **221** | chr2:178641241-178641315 | 0,858 | 0,889 | 0,845 | 0,903 |
| **222** | chr2:178640541-178640630 | 0,921 | 0,902 | 0,866 | 0,907 |
| **223** | chr2:178640048-178640110 | 0,927 | 0,895 | 0,892 | 0,904 |
| **224** | chr2:178639699-178639788 | 0,916 | 0,874 | 0,918 | 0,894 |
| **225** | chr2:178637369-178637419 | 0,794 | 0,839 | 0,625 | 0,498 |
| **226** | chr2:178636398-178636799 | 0,975 | 0,939 | 0,966 | 0,952 |
| **227** | chr2:178635963-178636241 | 0,971 | 0,937 | 0,954 | 0,942 |
| **228** | chr2:178635440-178635715 | 0,971 | 0,926 | 0,962 | 0,942 |
| **229** | chr2:178635165-178635304 | 0,969 | 0,919 | 0,958 | 0,931 |
| **230** | chr2:178634723-178634849 | 0,965 | 0,916 | 0,949 | 0,924 |
| **231** | chr2:178634366-178634629 | 0,968 | 0,916 | 0,954 | 0,933 |
| **232** | chr2:178633817-178634083 | 0,967 | 0,908 | 0,949 | 0,933 |
| **233** | chr2:178633413-178633676 | 0,968 | 0,915 | 0,943 | 0,939 |
| **234** | chr2:178633187-178633326 | 0,943 | 0,886 | 0,905 | 0,904 |
| **235** | chr2:178632918-178633044 | 0,950 | 0,893 | 0,922 | 0,916 |
| **236** | chr2:178632526-178632792 | 0,963 | 0,913 | 0,949 | 0,935 |
| **237** | chr2:178632147-178632413 | 0,959 | 0,903 | 0,950 | 0,915 |
| **238** | chr2:178631034-178631300 | 0,964 | 0,917 | 0,958 | 0,934 |
| **239** | chr2:178630804-178630943 | 0,964 | 0,930 | 0,953 | 0,944 |
| **240** | chr2:178630241-178630367 | 0,959 | 0,925 | 0,936 | 0,935 |
| **241** | chr2:178629301-178629443 | 0,960 | 0,923 | 0,923 | 0,892 |
| **242** | chr2:178625273-178625396 | 0,955 | 0,909 | 0,914 | 0,862 |
| **243** | chr2:178624465-178624731 | 0,978 | 0,944 | 0,455 | 0,420 |
| **244** | chr2:178622670-178622767 | 0,902 | 0,880 | 0,916 | 0,892 |
| **245** | chr2:178621840-178622008 | 0,974 | 0,931 | 0,975 | 0,965 |
| **246** | chr2:178621475-178621741 | 0,977 | 0,937 | 0,978 | 0,967 |
| **247** | chr2:178621102-178621368 | 0,977 | 0,937 | 0,977 | 0,965 |
| **248** | chr2:178620715-178620993 | 0,978 | 0,940 | 0,978 | 0,965 |
| **249** | chr2:178620217-178620625 | 0,978 | 0,934 | 0,980 | 0,959 |
| **250** | chr2:178619988-178620112 | 0,972 | 0,928 | 0,961 | 0,954 |
| **251** | chr2:178619621-178619887 | 0,973 | 0,939 | 0,976 | 0,956 |
| **252** | chr2:178618584-178618853 | 0,960 | 0,929 | 0,968 | 0,938 |
| **253** | chr2:178618189-178618491 | 0,969 | 0,938 | 0,975 | 0,958 |
| **254** | chr2:178617779-178618081 | 0,968 | 0,941 | 0,947 | 0,951 |
| **255** | chr2:178617325-178617512 | 0,960 | 0,906 | 0,955 | 0,935 |
| **256** | chr2:178617120-178617234 | 0,952 | 0,886 | 0,948 | 0,934 |
| **257** | chr2:178616729-178617013 | 0,964 | 0,906 | 0,952 | 0,949 |
| **258** | chr2:178616479-178616630 | 0,962 | 0,913 | 0,954 | 0,949 |
| **259** | chr2:178615641-178615788 | 0,956 | 0,903 | 0,921 | 0,943 |
| **260** | chr2:178615307-178615484 | 0,962 | 0,920 | 0,926 | 0,951 |
| **261** | chr2:178614847-178614968 | 0,954 | 0,921 | 0,877 | 0,940 |
| **262** | chr2:178614466-178614753 | 0,966 | 0,934 | 0,905 | 0,951 |
| **263** | chr2:178614052-178614348 | 0,974 | 0,934 | 0,898 | 0,961 |
| **264** | chr2:178613751-178613937 | 0,968 | 0,919 | 0,885 | 0,952 |
| **265** | chr2:178613161-178613276 | 0,963 | 0,914 | 0,887 | 0,949 |
| **266** | chr2:178612773-178613072 | 0,969 | 0,912 | 0,886 | 0,962 |
| **267** | chr2:178612277-178612576 | 0,972 | 0,933 | 0,870 | 0,961 |
| **268** | chr2:178612057-178612162 | 0,964 | 0,920 | 0,870 | 0,945 |
| **269** | chr2:178611758-178611954 | 0,969 | 0,923 | 0,880 | 0,955 |
| **270** | chr2:178611372-178611677 | 0,973 | 0,935 | 0,899 | 0,961 |
| **271** | chr2:178610993-178611271 | 0,971 | 0,926 | 0,889 | 0,955 |
| **272** | chr2:178610090-178610389 | 0,971 | 0,916 | 0,898 | 0,949 |
| **273** | chr2:178609684-178609986 | 0,971 | 0,924 | 0,922 | 0,945 |
| **274** | chr2:178609208-178609570 | 0,972 | 0,921 | 0,912 | 0,951 |
| **275** | chr2:178608606-178608908 | 0,970 | 0,916 | 0,899 | 0,948 |
| **276** | chr2:178608178-178608477 | 0,968 | 0,911 | 0,864 | 0,948 |
| **277** | chr2:178607785-178608081 | 0,969 | 0,909 | 0,871 | 0,947 |
| **278** | chr2:178607401-178607685 | 0,969 | 0,910 | 0,874 | 0,947 |
| **279** | chr2:178607021-178607314 | 0,969 | 0,917 | 0,886 | 0,946 |
| **280** | chr2:178605414-178605713 | 0,970 | 0,920 | 0,900 | 0,950 |
| **281** | chr2:178604987-178605295 | 0,966 | 0,907 | 0,882 | 0,948 |
| **282** | chr2:178604708-178604898 | 0,963 | 0,894 | 0,881 | 0,939 |
| **283** | chr2:178603876-178604305 | 0,961 | 0,903 | 0,881 | 0,942 |
| **284** | chr2:178602282-178602590 | 0,959 | 0,904 | 0,868 | 0,943 |
| **285** | chr2:178602002-178602150 | 0,948 | 0,874 | 0,848 | 0,928 |
| **286** | chr2:178601882-178601914 | 0,921 | 0,800 | 0,819 | 0,890 |
| **287** | chr2:178601658-178601787 | 0,939 | 0,871 | 0,853 | 0,910 |
| **288** | chr2:178601265-178601564 | 0,961 | 0,920 | 0,876 | 0,943 |
| **289** | chr2:178600854-178601171 | 0,963 | 0,928 | 0,880 | 0,946 |
| **290** | chr2:178599554-178599850 | 0,964 | 0,909 | 0,881 | 0,942 |
| **291** | chr2:178599146-178599445 | 0,940 | 0,884 | 0,863 | 0,926 |
| **292** | chr2:178598748-178599062 | 0,947 | 0,888 | 0,875 | 0,919 |
| **293** | chr2:178598506-178598654 | 0,916 | 0,817 | 0,855 | 0,875 |
| **294** | chr2:178597908-178598058 | 0,934 | 0,874 | 0,868 | 0,911 |
| **295** | chr2:178597538-178597819 | 0,967 | 0,915 | 0,899 | 0,948 |
| **296** | chr2:178595507-178595809 | 0,947 | 0,900 | 0,878 | 0,937 |
| **297** | chr2:178594344-178594646 | 0,951 | 0,896 | 0,887 | 0,945 |
| **298** | chr2:178593961-178594242 | 0,950 | 0,890 | 0,886 | 0,942 |
| **299** | chr2:178593568-178593867 | 0,953 | 0,899 | 0,887 | 0,946 |
| **300** | chr2:178593173-178593475 | 0,962 | 0,909 | 0,911 | 0,952 |
| **301** | chr2:178592775-178593083 | 0,959 | 0,911 | 0,899 | 0,954 |
| **302** | chr2:178592379-178592660 | 0,960 | 0,903 | 0,885 | 0,952 |
| **303** | chr2:178591978-178592277 | 0,961 | 0,910 | 0,871 | 0,953 |
| **304** | chr2:178591599-178591892 | 0,956 | 0,908 | 0,853 | 0,944 |
| **305** | chr2:178588538-178591504 | 0,963 | 0,915 | 0,871 | 0,947 |
| **306** | chr2:178587899-178588219 | 0,934 | 0,871 | 0,835 | 0,912 |
| **307** | chr2:178587516-178587800 | 0,935 | 0,873 | 0,850 | 0,917 |
| **308** | chr2:178587118-178587417 | 0,938 | 0,868 | 0,834 | 0,925 |
| **309** | chr2:178586505-178586807 | 0,937 | 0,888 | 0,851 | 0,922 |
| **310** | chr2:178585072-178585347 | 0,936 | 0,885 | 0,862 | 0,927 |
| **311** | chr2:178584669-178584968 | 0,934 | 0,888 | 0,826 | 0,928 |
| **312** | chr2:178584276-178584578 | 0,918 | 0,867 | 0,788 | 0,914 |
| **313** | chr2:178583607-178583906 | 0,927 | 0,849 | 0,845 | 0,911 |
| **314** | chr2:178582940-178583227 | 0,918 | 0,838 | 0,800 | 0,902 |
| **315** | chr2:178582296-178582592 | 0,920 | 0,847 | 0,801 | 0,901 |
| **316** | chr2:178581906-178582208 | 0,919 | 0,834 | 0,796 | 0,903 |
| **317** | chr2:178581499-178581804 | 0,923 | 0,834 | 0,791 | 0,902 |
| **318** | chr2:178580322-178580609 | 0,934 | 0,861 | 0,830 | 0,917 |
| **319** | chr2:178579939-178580229 | 0,928 | 0,835 | 0,824 | 0,908 |
| **320** | chr2:178579561-178579848 | 0,924 | 0,842 | 0,823 | 0,902 |
| **321** | chr2:178578806-178579393 | 0,921 | 0,865 | 0,817 | 0,899 |
| **322** | chr2:178578611-178578715 | 0,885 | 0,797 | 0,791 | 0,854 |
| **323** | chr2:178577988-178578185 | 0,918 | 0,831 | 0,820 | 0,900 |
| **324** | chr2:178577602-178577898 | 0,935 | 0,869 | 0,877 | 0,917 |
| **325** | chr2:178576923-178577510 | 0,924 | 0,868 | 0,868 | 0,900 |
| **326** | chr2:178576529-178576831 | 0,920 | 0,852 | 0,856 | 0,887 |
| **327** | chr2:178559311-178576416 | 0,968 | 0,926 | 0,932 | 0,951 |
| **328** | chr2:178558341-178558637 | 0,935 | 0,817 | 0,868 | 0,894 |
| **329** | chr2:178557648-178558235 | 0,942 | 0,848 | 0,877 | 0,902 |
| **330** | chr2:178557253-178557555 | 0,948 | 0,854 | 0,872 | 0,911 |
| **331** | chr2:178556848-178557144 | 0,935 | 0,825 | 0,861 | 0,893 |
| **332** | chr2:178554865-178555152 | 0,925 | 0,816 | 0,854 | 0,884 |
| **333** | chr2:178554453-178554752 | 0,935 | 0,843 | 0,884 | 0,898 |
| **334** | chr2:178553914-178554216 | 0,939 | 0,852 | 0,890 | 0,902 |
| **335** | chr2:178553502-178553807 | 0,936 | 0,859 | 0,885 | 0,898 |
| **336** | chr2:178551630-178553396 | 0,936 | 0,875 | 0,890 | 0,894 |
| **337** | chr2:178550967-178551260 | 0,920 | 0,837 | 0,880 | 0,873 |
| **338** | chr2:178549986-178550273 | 0,914 | 0,833 | 0,870 | 0,876 |
| **339** | chr2:178549570-178549869 | 0,931 | 0,861 | 0,880 | 0,900 |
| **340** | chr2:178547407-178549473 | 0,943 | 0,884 | 0,878 | 0,907 |
| **341** | chr2:178547003-178547305 | 0,937 | 0,876 | 0,863 | 0,902 |
| **342** | chr2:178546600-178546905 | 0,935 | 0,867 | 0,858 | 0,903 |
| **343** | chr2:178546212-178546502 | 0,935 | 0,866 | 0,860 | 0,900 |
| **344** | chr2:178545820-178546116 | 0,943 | 0,885 | 0,878 | 0,910 |
| **345** | chr2:178545388-178545693 | 0,940 | 0,894 | 0,880 | 0,912 |
| **346** | chr2:178544201-178544506 | 0,933 | 0,880 | 0,860 | 0,906 |
| **347** | chr2:178543834-178544115 | 0,930 | 0,875 | 0,845 | 0,908 |
| **348** | chr2:178543069-178543662 | 0,943 | 0,903 | 0,849 | 0,911 |
| **349** | chr2:178542662-178542949 | 0,944 | 0,891 | 0,869 | 0,908 |
| **350** | chr2:178542264-178542563 | 0,949 | 0,888 | 0,899 | 0,904 |
| **351** | chr2:178541282-178541584 | 0,951 | 0,901 | 0,897 | 0,915 |
| **352** | chr2:178540068-178540370 | 0,953 | 0,908 | 0,885 | 0,926 |
| **353** | chr2:178539382-178539966 | 0,955 | 0,912 | 0,884 | 0,929 |
| **354** | chr2:178538946-178539251 | 0,957 | 0,914 | 0,890 | 0,935 |
| **355** | chr2:178538540-178538839 | 0,965 | 0,921 | 0,890 | 0,953 |
| **356** | chr2:178537342-178537917 | 0,965 | 0,941 | 0,913 | 0,950 |
| **357** | chr2:178536938-178537243 | 0,870 | 0,887 | 0,758 | 0,857 |
| **358** | chr2:178535982-178536575 | 0,900 | 0,890 | 0,782 | 0,893 |
| **359** | chr2:178530241-178535849 | 0,942 | 0,942 | 0,881 | 0,948 |
| **360** | chr2:178529960-178530116 | 0,904 | 0,917 | 0,832 | 0,924 |
| **361** | chr2:178528528-178529219 | 0,935 | 0,941 | 0,912 | 0,940 |
| **362** | chr2:178528274-178528427 | 0,935 | 0,938 | 0,912 | 0,939 |
| **363** | chr2:178527446-178527748 | 0,886 | 0,880 | 0,911 | 0,944 |
| **364** | chr2:178525989-178527307 | 0,901 | 0,928 | 0,857 | 0,928 |

**Table S4.** Published cases in the literature with longitudinal clinical info, fitting the expected disease course

| **Var 1** | **Var 2** | **Genotype** | **Publication** | **Exon var 1** | **Exon usage fetal skeletal muscles 1** | **Exon usage adult skeletal muscles 1** | **Exon var 2** | **Exon usage fetal skeletal muscles 2** | **Exon usage adult skeletal muscles 2** | **Improving/stable/worsening** |
| --- | --- | --- | --- | --- | --- | --- | --- | --- | --- | --- |
| c.38661_38665del (p.Lys12887AsnfsTer6) | c.38661_38665del p.(Lys12887AsnfsTer6) | hom | Fernández-Marmiesse, Ana et al. (2017), doi:10.1016/j.nmd.2016.11.002 | 198 | **11,0%** | **0,9%** | 198 | **11,0%** | **0,9%** | improving |
| c.105109_105110del (p.Thr35037HisfsTer9) | c.37237_37240del (p.Lys12413CysfsTer533) | comp hetz | Oates, Emily C et al. (2018), doi:10.1002/ana.25241 | 359 | 94,2% | 94,2% | 181 | **6,7%** | **1,1%** | improving |
| c.35794G>T (p.Glu11932Ter) | c.22973C>A (p.Ser7658Ter) | comp hetz | Oates, Emily C et al. (2018), doi:10.1002/ana.25242 | 163 | **39,4%** | **0,9%** | 80 | 96,3% | 85,0% | improving: indipendent walking acquired |
| c.35794G>T (p.Glu11932Ter) | c.7639del (p.Thr2547LeufsTer43) | comp hetz | Oates, Emily C et al. (2018), doi:10.1002/ana.25243 | 163 | **39,4%** | **0,9%** | 34 | 99,2% | 97,3% | improving: indipendent walking being acquired |
| c.35794G>T (p.Glu11932Ter) | c.7639del (p.Thr2547LeufsTer43) | comp hetz | Oates, Emily C et al. (2018), doi:10.1002/ana.25244 | 163 | **39,4%** | **0,9%** | 34 | 99,2% | 97,3% | improving: indipendent walking acquired |
| c.40004_40008del (p.Val13335GlufsTer22) | c.40004_40008del p.(Val13335GlufsTer22) | hom | Kasinathan, Ananthanarayanan et al. (2018), doi:10.1097/CND.0000000000000167 | 214 | **77,3%** | **39,3%** | 214 | **77,3%** | **39,3%** | stable/improving (17 months old, not walking but she is able to hold a spoon, few info) |
| c.80850C>G (p.Tyr26950Ter) | c.35794G>T (p.Glu11932Ter) | comp hetz | Oates, Emily C et al. (2018), doi:10.1002/ana.25241 | 327 | 96,8% | 92,6% | 163 | **39,4%** | **0,9%** | improving: indipendent walking acquired |
| c.98506C>T (p.Arg32836Ter) | c.35794G>T (p.Glu11932Ter) | comp hetz | Oates, Emily C et al. (2018), doi:10.1002/ana.25242 | 353 | 95,5% | 91,2% | 163 | **39,4%** | **0,9%** | alive at 8 yrs old, few info |
| c.79684C>T (p.Arg26562Ter) | c.37261A>T p.(Lys12421Ter) | comp hetz | Savarese, Marco et al. (2020), doi:10.1038/s41436-020-0914-2 | 327 | 96,8% | 92,6% | 181 | **6,7%** | **1,1%** | alive at 10 yrs, few info |
| c.36122del (p.Pro12041LeufsTer117) | c.36122del (p.Pro12041LeufsTer117) | hom | Chervinsky, Elena et al. (2018) doi:10.1002/ajmg.a.38639 | 168 | **60,5%** | **9,0%** | 168 | **60,5%** | **9,0%** | perinatal lethality |
| c.36122del (p.Pro12041LeufsTer117) | c.36122del (p.Pro12041LeufsTer117) | hom | Chervinsky, Elena et al. (2018) doi:10.1002/ajmg.a.38640 | 168 | **60,5%** | **9,0%** | 168 | **60,5%** | **9,0%** | fetal lethality |
| c.36122del (p.Pro12041LeufsTer117) | c.36122del (p.Pro12041LeufsTer117) | hom | Chervinsky, Elena et al. (2018) doi:10.1002/ajmg.a.38641 | 168 | **60,5%** | **9,0%** | 168 | **60,5%** | **9,0%** | fetal lethality |
| c.36122del (p.Pro12041LeufsTer117) | c.36122del (p.Pro12041LeufsTer117) | hom | Chervinsky, Elena et al. (2018) doi:10.1002/ajmg.a.38642 | 168 | **60,5%** | **9,0%** | 168 | **60,5%** | **9,0%** | fetal lethality |
| c.36122del (p.Pro12041LeufsTer117) | c.36122del (p.Pro12041LeufsTer117) | hom | Chervinsky, Elena et al. (2018) doi:10.1002/ajmg.a.38643 | 168 | **60,5%** | **9,0%** | 168 | **60,5%** | **9,0%** | postnatal death (< 1 year of age) |
| c.36122del (p.Pro12041LeufsTer117) | c.36122del (p.Pro12041LeufsTer117) | hom | Chervinsky, Elena et al. (2018) doi:10.1002/ajmg.a.38644 | 168 | **60,5%** | **9,0%** | 168 | **60,5%** | **9,0%** | postnatal death (< 1 year of age) |
| c.36122del (p.Pro12041LeufsTer117) | c.36122del (p.Pro12041LeufsTer117) | hom | Chervinsky, Elena et al. (2018) doi:10.1002/ajmg.a.38645 | 168 | **60,5%** | **9,0%** | 168 | **60,5%** | **9,0%** | postnatal death (< 1 year of age) |
| c.36122del (p.Pro12041LeufsTer117) | c.36122del (p.Pro12041LeufsTer117) | hom | Chervinsky, Elena et al. (2018) doi:10.1002/ajmg.a.38646 | 168 | **60,5%** | **9,0%** | 168 | **60,5%** | **9,0%** | postnatal death (< 1 year of age) |
| c.36122del (p.Pro12041LeufsTer117) | c.36122del (p.Pro12041LeufsTer117) | hom | Chervinsky, Elena et al. (2018) doi:10.1002/ajmg.a.38647 | 168 | **60,5%** | **9,0%** | 168 | **60,5%** | **9,0%** | postnatal death (< 1 year of age) |
